# Urinary Excretion of Mercapturic Acids of the Rodent Carcinogen Methyleugenol after a Single Meal of Basil Pesto: A Controlled Exposure Study in Humans

**DOI:** 10.1021/acs.chemrestox.3c00212

**Published:** 2023-10-24

**Authors:** Kai Nieschalke, Nick Bergau, Sönke Jessel, Albrecht Seidel, Susanne Baldermann, Monika Schreiner, Klaus Abraham, Alfonso Lampen, Bernhard H. Monien, Burkhard Kleuser, Hansruedi Glatt, Fabian Schumacher

**Affiliations:** † Department of Nutritional Toxicology, Institute of Nutritional Science, University of Potsdam, 14558 Nuthetal, Germany; ‡ Department of Food Safety, German Federal Institute for Risk Assessment (BfR), 10589 Berlin, Germany; § Department of Pharmacology and Toxicology, Institute of Pharmacy, Freie Universität Berlin, 14195 Berlin, Germany; ∥ Biochemical Institute for Environmental Carcinogens, Prof. Dr. Gernot Grimmer-Foundation, 22927 Grosshansdorf, Germany; ⊥ Department Plant Quality and Food Security, Leibniz Institute of Vegetable and Ornamental Crops (IGZ), 14979 Grossbeeren, Germany; # Faculty of Life Sciences: Food, Nutrition & Health, University of Bayreuth, 95326 Kulmbach, Germany

## Abstract

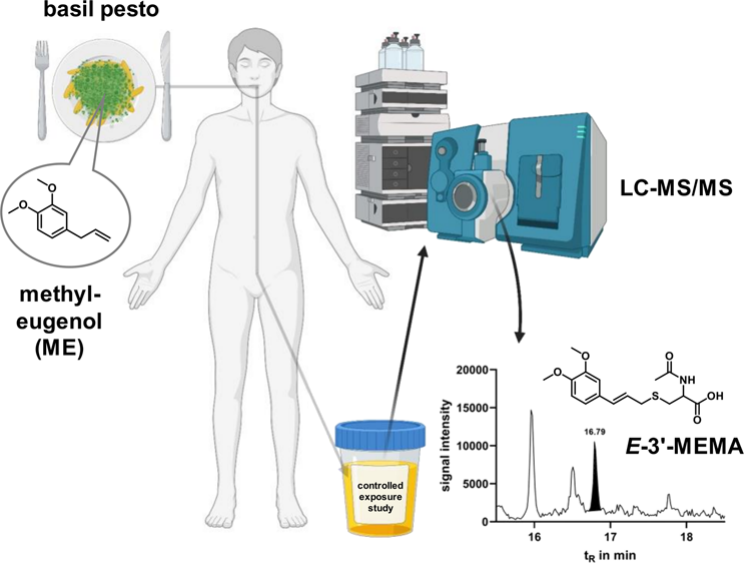

Methyleugenol (ME), found in numerous plants and spices, is a rodent carcinogen and is classified as “possibly carcinogenic to humans”. The hypothesis of a carcinogenic risk for humans is supported by the observation of ME-derived DNA adducts in almost all human liver and lung samples examined. Therefore, a risk assessment of ME is needed. Unfortunately, biomarkers of exposure for epidemiological studies are not yet available. We hereby present the first detection of *N*-acetyl-l-cysteine conjugates (mercapturic acids) of ME in human urine samples after consumption of a popular ME-containing meal, pasta with basil pesto. We synthesized mercapturic acid conjugates of ME, identified the major product as *N*-acetyl-*S*-[3′-(3,4-dimethoxyphenyl)allyl]-l-cysteine (*E*-3′-MEMA), and developed methods for its extraction and LC–MS/MS quantification in human urine. For conducting an exposure study in humans, a basil cultivar with a suitable ME content was grown for the preparation of basil pesto. A defined meal containing 100 g of basil pesto, corresponding to 1.7 mg ME, was served to 12 participants, who collected the complete urine at defined time intervals for 48 h. Using *d*_6_-*E*-3′-MEMA as an internal standard for LC–MS/MS quantification, we were able to detect *E*-3′-MEMA in urine samples of all participants collected after the ME-containing meal. Excretion was maximal between 2 and 6 h after the meal and was completed within about 12 h (concentrations below the limit of detection). Excreted amounts were only between 1 and 85 ppm of the ME intake, indicating that the ultimate genotoxicant, 1′-sulfooxy-ME, is formed to a subordinate extent or is not efficiently detoxified by glutathione conjugation and subsequent conversion to mercapturic acids. Both explanations may apply cumulatively, with the ubiquitous detection of ME DNA adducts in human lung and liver specimens arguing against an extremely low formation of 1′-sulfooxy-ME. Taken together, we hereby present the first noninvasive human biomarker reflecting an internal exposure toward reactive ME species.

## Introduction

Methyleugenol (ME) is a secondary metabolite that is present in a huge variety of plants, spices, and essential oils.^1,2^ It is a genotoxic carcinogen in rodents^3^ and was classified as “possibly carcinogenic to humans” (Group 2B) by the International Agency for Research on Cancer (IARC).^4^

ME induced gene and deletion mutations in the liver of transgenic gpt delta mice.^5^ Mutations in β-catenin were found as an early event in liver tumors of mice treated with ME.^6^ Furthermore, ME increased the mutational burden in hepatocellular carcinomas of mice in a dose-dependent manner,^7^ leading to mutational signatures similar to those in human hepatocellular carcinomas with known exposure to the carcinogens aflatoxin or benzo[*a*]pyrene.^8^ In a recent study, it was shown that ME caused DNA damage-dependent replication stress resulting in mitochondrial apoptosis via the p53-Bax pathway.^9^

ME-derived DNA adducts, found in cell culture experiments,^10,11^ were also detected in mice,^12−15^ and, most importantly, in humans.^16−18^ To be precise, the presence of DNA adducts, primarily *N*^2^-(methylisoeugenol-3′-yl)-2′-deoxyguanosine, was demonstrated in 150 out of 151 surgical human liver samples^16,17^ and 10 out of 10 lung samples^18^ investigated. The proof of these adducts in nearly all human tissue samples examined is rather unique for DNA adducts formed by xenobiotics. This observation emphasizes the genotoxic potential and, thus, the risk emanating from ME. To address this issue, more research and data, especially from epidemiological studies, are needed. Biomarkers reflecting exposure to ME and its active metabolites would be very helpful in such studies.

A biomarker of exposure can be defined as a xenobiotic, its metabolites or reaction products with target molecules that can be measured in a certain compartment or fluid of an organism.^19^ Regarding this definition, an ideal biomarker of exposure for risk assessment especially in terms of the carcinogenic potential of ME should be associated with the metabolic activation of this phytochemical.

**Scheme 1:**
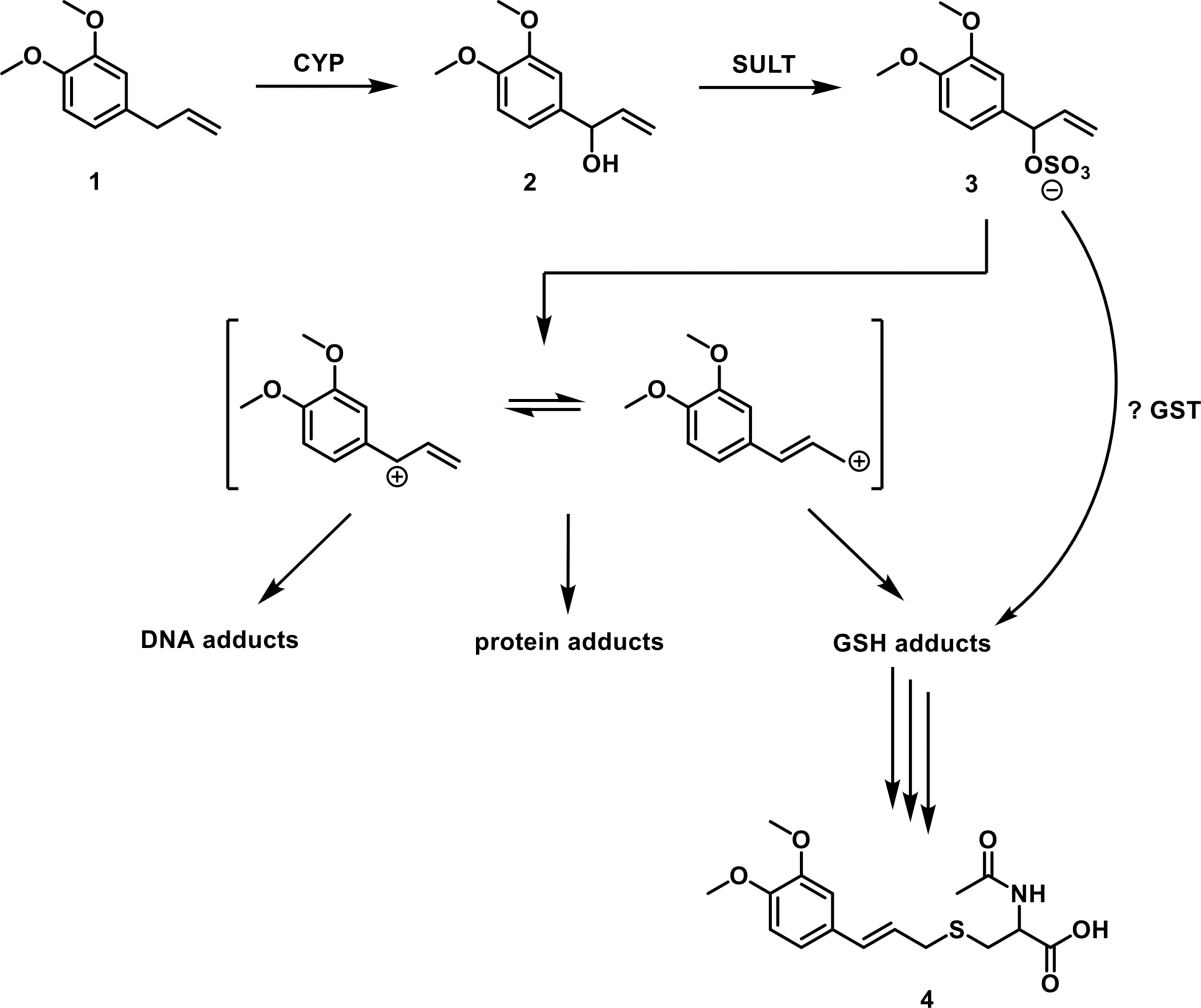
Toxification of ME ME (1) is converted after CYP-mediated hydroxylation (1′-hydroxy-ME (2)) and subsequent SULT-dependent sulfonation to 1′-sulfooxy-ME (3), an electrophile, able to form adducts with cellular nucleophiles, e.g., DNA, proteins, and GSH, probably in an Sn1 reaction (i.e., via a distinct carbocation). Potentially, the GSH adduct may also be formed by GSTs directly from 1′-sulfooxy-ME. Further processing of the GSH adducts (and possibly some protein adducts) yields isomeric *N*-acetyl-l-cysteine conjugates (mercapturic acids) that are excreted in the urine. The most abundant mercapturic acid, identified as the *E* form of *N*-acetyl-*S*-[3′-(3,4-dimethoxyphenyl)allyl]-l-cysteine (*E*-3′-MEMA (4)), was quantified in the present study in human urine, using a chemically prepared pure standard. Two further isomers, putatively 1′-(*R*) and 1′-(*S*) diastereomers of *N*-acetyl-*S*-[1′-(3,4-dimethoxyphenyl)allyl]-l-cysteine were also detected in some urine samples. Due to the weakness of their signals in urine and the lack of purified standards, they were not quantified.

The metabolite 1′-hydroxy-ME was more potent than ME in the induction of hepatomas and the formation of DNA adducts in mouse liver.^12,20^ Genetic knockout of sulfotransferase (SULT) 1A1 reduced DNA adduct formation by ME in mouse liver by 99%. Likewise, the induction of unscheduled DNA synthesis (UDS) by ME in primary cultures of rat hepatocytes was fully suppressed in the presence of the SULT1 inhibitor pentachlorophenol.^3^ These and other findings (including the chemical structure of the DNA adducts detected) indicate that the mechanism underlying the toxification of ME is similar to that of other allylalkoxybenzenes, i.e., estragole or safrole,^21^ congeners extensively studied by the group of Miller.^22^ In short, cytochrome P450 (CYP)-mediated hydroxylation of the benzylic carbon, followed by sulfonation via SULTs generates a reactive sulfuric acid ester, which, after the loss of a sulfate moiety, leads to a highly reactive ME carbocation. This electrophilic species is thought to be mainly responsible for adduct formation (Scheme 1).

In detail, the metabolism of ME in microsomal systems has been extensively investigated in two laboratories. In laboratory A, ME (100 and 500 μM) was incubated with liver microsomes from rats (control and Aroclor-treated), cattle, and humans (150 gender-mixed donors) in a first study.^11^ In a second study,^12^ the metabolism of ME (200 μM) was investigated using hepatic microsomes from mice strains, differing in their SULT1A1 status. In these 12 experimental settings, ring hydroxylation (leading to the formation of 6-hydroxy-ME) and *O*-demethylation (generating eugenol and chavibetol) contributed 0–27.4 and 0–13.8%, respectively, to the metabolism (sum all metabolites; all calculations were made by us from the published data, using molar units). The remaining metabolites involved oxidation of the allyl group; taken together, they clearly dominated the biotransformation of ME (71.9–98.7%). Generally, 1′-hydroxy-ME was the most abundant individual ME metabolite (36.4–69.4%), often followed by 3′-hydroxymethylisoeugenol (3′-hydroxy-MIE, 10.4–42.4%). The corresponding aldehyde, 3′-oxo-MIE, added 0–6.8% to the metabolism of ME. Epoxidation of the allylic double bond accounted for 0–16.2% of the metabolism. Luo et al.^23^ have shown that the 2′,3′-allylic epoxides of allylbenzene, estragole, eugenol, and safrole are good substrates for microsomal epoxide hydrolase. Therefore, only the resulting 2′,3′-dihydroxy-2′,3′-dihydro-ME was detected with rat, bovine, and human microsomes. However, with microsomes from the mouse, a species showing particularly low hepatic expression of microsomal epoxide hydrolase,^24^ both the epoxide and the dihydrodiol were detected, in a ratio of nearly 1:2.

The microsomal metabolism studies in laboratory B^25,26^ involved more different substrate concentrations than those in laboratory A, enabling the determination of apparent *V*_max_ and *K*_m_ values. Hepatic microsomes from male and female Sprague–Dawley and Fischer 344 rats as well as pooled gender-mixed human liver microsomes were used. When *V*_max_ values were used for calculating the contribution of the different metabolic pathways in these five models, 1′-hydroxy-ME was the most abundant individual ME metabolite (36.2–50.1%). The other metabolites detected were 3′-hydroxy-MIE (10.3–12.9%), 6-hydroxy-ME (3.9–4.7%), the *O*-demethylation products eugenol and chavibetol (0.9–6.7%), and 2′,3′-dihydroxy-2′,3′-dihydro-ME (21.8–44.0%) as well as an unknown metabolite (M6), detected only with rat microsomes (0.9–6.7%). Unlike in the studies of laboratory A, 3′-oxo-MIE was not detected in laboratory B, probably due to the addition of an antioxidant, ascorbic acid, to the incubation mixture. Apart from the formation of M6 in rat microsomes, the profile of metabolites was rather similar with hepatic microsomes from the different sources (male and female rats, humans), if *V*_max_ values were used for the calculation (reflecting the situation at high substrate concentrations). This situation changed if the calculation was based on the catalytic efficiency (*V*_max_/*K*_m_), depicting the conditions at low substrate concentrations. This led to substantial shifts in the metabolite profiles. These shifts were different in male rats (strong increase in 3′-hydroxy-MIE at the expense of 1′-hydroxy-ME), female rats (strong increase in 3′-hydroxy-MIE at the expense of 2′,3′-dihydroxy-2′,3′-dihydro-ME), and humans (strong increase in 2′,3′-dihydroxy-2′,3′-dihydro-ME at the expense of 1′-hydroxy-ME and 3′-hydroxy-MIE).

The metabolism of ME and its isomer, MIE, was also studied in rats in vivo.^27^ Urine collected over a period of 24 h after oral administration of ME (200 mg/kg) contained a total of ten metabolites (each accounting for ≥1% of the dose); together, they represented 95% of the dose. Among the microsomal metabolites described in the preceding sections, only 6-hydroxy-ME was detected in native urine (2% of the dose). In human liver microsomes, it contributed 0–3.4% to the metabolism of ME, less than that in liver microsomes from other species [calculated from data published by Cartus et al.^11^ and Al-Subeihi et al.^26^]. For this reason, and as 6-hydroxy-ME is not involved in bioactivation, it is not suited as a biomarker. Eugenol and chavibetol were found in urine treated with glucuronidase/sulfatase at levels of 7 and 4% of the dose, respectively. Obviously, these metabolites are not suited as biomarkers for exposure to ME, as they may also be found after exposure to eugenol and chavibetol. Five other metabolites, 3,4-dimethoxybenzoic acid (2%), dimethoxycinnamic acid (2%), 3-hydroxy-3-(3,4-dimethoxyphenyl)propionic acid (2%), 3,4-dimethoxybenzoylglycine (30%), and 3,4-dimethoxycinnamoylglycine (24%) were detected at similar levels in urine of rats treated with the noncarcinogenic congener, MIE, and therefore are useless as biomarkers for ME exposure and activation. 3,4-Dimethylphenylacetic acid (3%) also represents a metabolite of the neurotransmitter dopamine (formed by monoaminoxidase and aldehyde dehydrogenase). Finally, 2-hydroxy-3-(3,4-dimethoxyphenyl)propionic acid (20%) is probably formed via 3′-oxidation of 2′,3′-dihydroxy-2′,3′-dihydro-ME. This metabolic pathway does not involve sulfo conjugation, in contrast to the DNA adduct formation and other genotoxic effects of ME. Thus, none of the urinary metabolites described by Solheim and Scheline^27^ is suitable as a biomarker for exposure to ME and its active metabolites. Although in this study, 95% of the dose of ME was recovered as metabolites in urine, metabolites were additionally detected in bile. In particular, in bile treated with glucuronidase/sulfatase 1′-hydroxy-ME was found at relatively high levels. Probably it was released from its glucuronide since 1′-sulfooxy-ME is very short-lived. Glutathione (GSH) conjugates and other metabolites of the mercapturic acid pathways were not detected in that study; however, the method used (extraction of the acidified urine or bile with ether followed by gas chromatography) was not suitable for their detection.

Besides DNA adducts, additional reaction products of ME with typical nucleophilic cell targets have been described in the literature. For instance, liver protein adducts were demonstrated in ME-treated rats via ELISA and immunoblotting employing antisera recognizing the 3,4-dimethoxyphenyl moiety of ME.^28,29^ In this context, a dose-dependent formation of an adduct with a 44 kDa protein was observed, but no further information concerning its structure was provided. Liver protein adducts were also found in mice receiving ME.^30^ Here, the presented l-cysteine conjugates of ME were affiliated to the ME carbocation, ME-2′,3′-oxide, and (*E*)-3′-oxo-MIE. Interestingly, the latter metabolite has been shown to inhibit human topoisomerase I activity in vitro.^31^ Whether this could be due to protein adduction is not known. Besides DNA and protein adducts, ME conjugates of GSH and l-cysteine were reported in bile and urine of rats after ME administration.^32^ Furthermore, a nucleoside adduct, *N*^6^-(methylisoeugenol-3′-yl)-2′-deoxyadenosine, was shown to be excreted in urine of ME-fed rats in a dose- and time-dependent manner.^33^

Taken together, adducts of metabolically activated ME with cellular nucleophiles have been described for DNA, liver proteins, GSH, and some of their degradation products. Nonetheless, nearly all published ME adducts were solely identified, if at all, in animal studies where either high dosages of ME for treatment were used or the route of administration differed from the usual exposure scenario via food. DNA adducts of ME are the only species that were also found in human tissue samples. For their detection, however, surgery is necessary. Moreover, DNA adduct levels do not reflect the actual or cumulative exposure to a genotoxic substance because they may persist for varying time periods before their elimination by repair or cell turnover.^34^ Thus, there is a need for novel biomarkers of exposure toward ME, which can be used for risk assessment.

We here present the results of an exposure study in humans in which we were able to detect mercapturic acid conjugates of ME (MEMA) in urine of volunteers who consumed a typical ME-containing meal (basil pesto with pasta). MEMA was detected in urine samples from all participants. To the best of our knowledge, this is the first report on the determination of mercapturic acid conjugates of ME and, more importantly, the first proof of a metabolite resulting from metabolic activation of ME, in humans.

## Experimental Procedures

### Chemicals

Acetone, *N*-acetylcysteine (NAC) methyl ester (≥90%), cyclohexane, dichloromethane (DCM), eugenol (99%), *d*_3_-iodomethane (≥99.5 atom % D), tetrahydrofuran (THF), trimethylamine, and vinylmagnesium bromide (1 M in THF) were purchased from Sigma-Aldrich (Taufkirchen, Germany) and used without further purification. Unless stated otherwise, all other reagents (p.a.) were purchased from Sigma-Aldrich. HPLC-grade acetonitrile, methanol, ethyl acetate (EtOAc), tributylamine, acetic acid, and silica gel 60 (0.063–0.200 mm) were obtained from Merck (Darmstadt, Germany). *E*-3′-MEMA was synthesized by Chiroblock GmbH (Bitterfeld-Wolfen, Germany). HPLC-grade water was prepared by using a Milli-Q Integral Water Purification System from Millipore Merck (Darmstadt, Germany).

### Synthesis of MEMA Isomer Mixture

The MEMA isomer mixture was synthesized starting with the conversion of 3,4-dimethoxybenzaldehyde to 1′-hydroxy-ME. Here, under argon atmosphere, a solution of 3,4-dimethoxybenzaldehyde (166 mg, 1.00 mmol) in 5 mL of dry THF was added dropwise to a 1 M solution of vinylmagnesium bromide in dry THF (1.2 mL). After stirring for 2 h at room temperature, the reaction was quenched with 5 mL of saturated ammonium chloride solution, and the mixture was extracted with EtOAc (3 × 5 mL). The combined organic phases were dried over Na_2_SO_4_, filtered, and concentrated. The crude product was purified by column chromatography on silica using a mixture of cyclohexane and EtOAc (2:1, v/v) to afford 1′-hydroxy-ME as a yellow oil (140 mg, yield 72%). NMR (500 MHz, CDCl_3_): δ 6.96–6.88 (m, H-*arom.*, 2H), 6.85 (d, *J* = 8.1 Hz, 1H), 6.06 (ddd, H-2′, *J* = 17.1 Hz, *J* = 10.3 Hz, *J* = 5.8 Hz, 1H), 5.37 (dt, H-3′a, *J* = 17.1 Hz, *J* = 1.4 Hz, 1H), 5.20 (dt, H-3′b, *J* = 10.3 Hz, 1H), 5.19–5.15 (m, H-1′, 1H), 3.89 (s, O–C*H*_*3*_, 3H), 3.88 (s, O–C*H*_*3*_, 3H).

The following step included the reaction of 1′-hydroxy-ME to *N*-acetyl-*S*-(1′-(3,4-dimethoxyphenyl)allyl)-l-cysteine methyl ester. Therefore, methanesulfonyl chloride (67 μL, 99 mg, 864 μmol) was added to a solution of 1′-hydroxy-ME (140 mg, 720 mmol) and trimethylamine (121 μL, 87 mg, 864 μmol) in 5 mL of dry THF at room temperature. Subsequently, a mixture of NAC methyl ester (154 mg, 870 μmol) and trimethylamine (121 μL, 87 mg, 864 μmol) in 5 mL of dry THF was added dropwise directly to this suspension. After stirring for 2 h at room temperature, the reaction mixture was filtered, and the solvent was removed under reduced pressure. The crude product was purified by column chromatography on silica using a mixture of DCM and methanol (9:1, v/v) to give *N*-acetyl-*S*-(1′-(3,4-dimethoxyphenyl)allyl)-l-cysteine methyl ester as a pale yellow solid (132 mg, yield 52%). The resulting product consisted of a mixture of stereo- and regio-isomers that was used as such for the subsequent step without further separation and purification of isomers.

**Figure 1:**
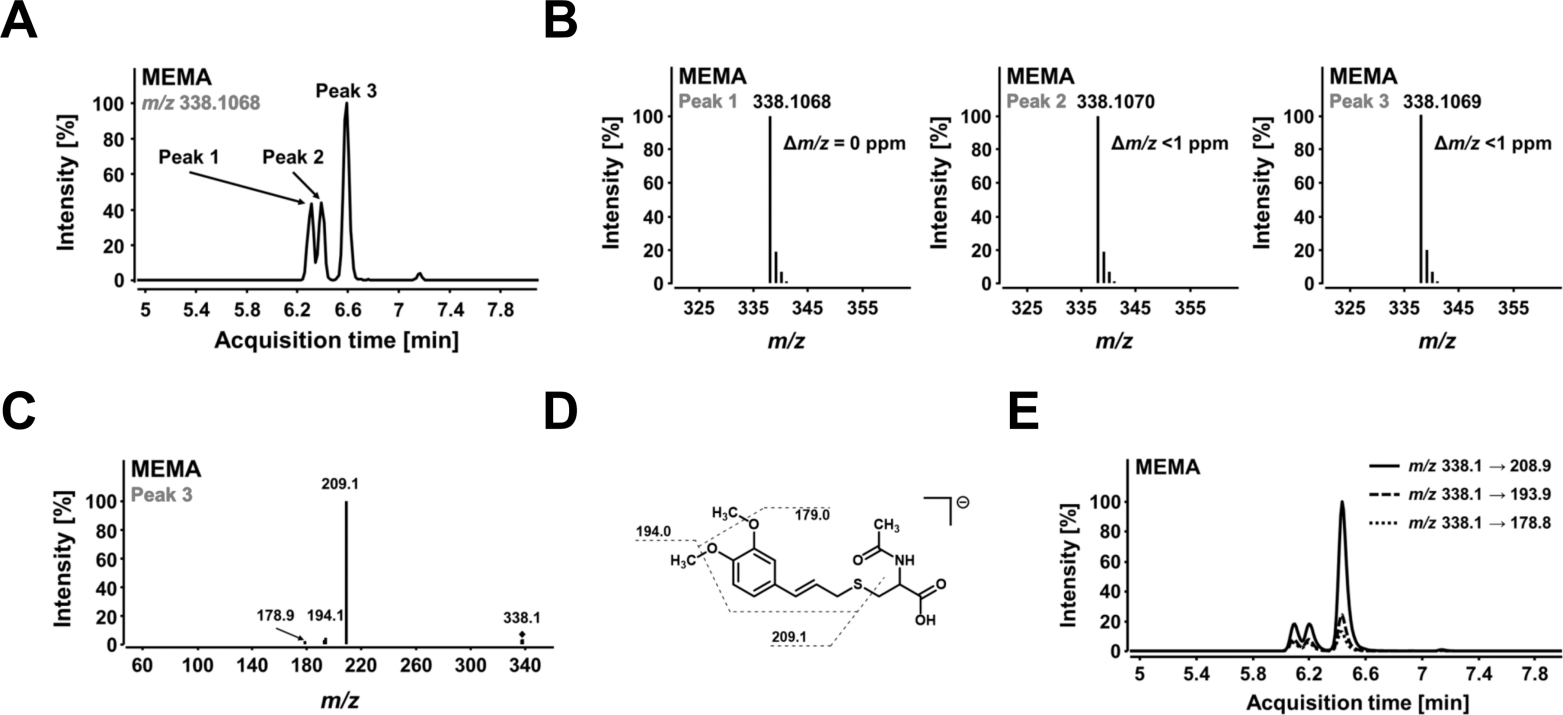
LC–MS characterization of the synthesized MEMA isomer mixture using instrumental setup “system 1”. (A) High-resolution mass spectrometry (HRMS) chromatogram of MEMA obtained in ESI- single ion monitoring (SIM) mode. Three prominent signals (Peaks 1, 2, and 3) were detected at *m*/*z* 338.1068. (B) Isotopic pattern of isomeric MEMA peaks and corresponding mass error (Δ*m*/*z*). (C) Product ion mass spectrum of Peak 3 obtained at a collision energy of 12 eV. The associated precursor ion was set at *m*/*z* 338.1. Product ion mass spectra of Peaks 1 and 2 are depicted in Suppl. Figure S1. (D) Chemical structure of *E*-3′-MEMA and suspected fragmentation pattern. (E) Multiple reaction monitoring (MRM) chromatogram of MEMA. An overlay of the different transitions used for analysis is shown.

Lithium hydroxide monohydrate (19 mg, 450 μmol) was added to a solution of *N*-acetyl-*S*-(1-(3,4-dimethoxyphenyl)allyl)-l-cysteine methyl ester (132 mg, 374 μmol) in 6 mL of water/MeOH (1:2, v/v). After stirring for 5 h at room temperature, the solvents were removed under reduced pressure, and the crude material was purified by column chromatography on silica using a mixture of DCM and methanol (4:1, v/v) to give *N*-acetyl-*S*-(1′-(3,4-dimethoxyphenyl)allyl)-l-cysteine as a pale yellow solid (78 mg, yield 62%). The purified product consisted of a mixture of three stereo- and regio-isomers that could not be separated for NMR spectroscopy in the preparative scale. However, they were characterized by LC–MS/MS analysis (Figures 1 and S1). Of note, the MEMA isomer mixture was applied for extraction optimization and LC–MS/MS method development only but not as a reference standard in the human exposure study. Instead, the reference material of the most prominent isomer, *E*-3′-MEMA, was separately synthesized by Chiroblock GmbH. ^1^H NMR, ^13^C NMR, and MS spectra are given in Supplementary Figure S2.

### Synthesis of *d*_6_-MEMA

*d*_6_-MEMA was synthesized as described for MEMA with an additional step including the synthesis of *d*_6_-3,4-dimethoxybenzaldehyde as a stable isotopically labeled starting material. Therefore, potassium carbonate (553 mg, 4.00 mmol) was added to a solution of 3,4-dihydroxybenzaldehyde (138 mg, 1.00 mmol) and *d*_3_-iodomethane (174 mg, 1.20 mmol) in 20 mL of acetone. The reaction mixture was refluxed for 6 h, cooled to room temperature, and filtered. The solvent was removed under reduced pressure, and the resulting oil was purified by column chromatography on silica using a mixture of cyclohexane and EtOAc (2:1, v/v) to give *d*_6_-3,4-dimethoxybenzaldehyde as a colorless oil (145 mg, yield 84%). The product was utilized for the subsequent synthesis and purification of *d*_6_-MEMA (same route as for MEMA), which was obtained as a pale-yellow solid (59 mg, yield 63%). The purified product consisted of a mixture of stereo- and positional isomers, which was not suitable for NMR spectroscopy and therefore characterized by LC–MS/MS analysis (Suppl. Figure S3). Quantification of the most prominent isomer, *d*_6_-*E*-3′-MEMA (to be applied as an internal standard in the human exposure study) was achieved by comparing mass spectrometric signal intensities to the isomerically pure *E*-3′-MEMA reference standard (Supplementary Figure S2).

### Synthesis of *d*_3_-ME

A mixture of eugenol (164 mg, 1.00 mmol), *d*_3_-iodomethane (363 mg, 2.50 mmol), and potassium carbonate (276 mg, 2.00 mmol) in 10 mL of acetone was refluxed for 6 h. The pale-yellow suspension was cooled and filtered. After the removal of the solvent under reduced pressure, the resulting oil was purified by column chromatography on silica using a mixture of cyclohexane and EtOAc (3:1, v/v) to give *d*_3_-ME as a colorless oil (166 mg, yield 92%). The purified product was characterized by GC–MS/MS analysis (Supplementary Figure S4).

### Selection and Cultivation of Basil Cultivar

Twenty seeds each of 18 different cultivars of basil *Ocimum basilicum* (Suppl. Table S1) were purchased from Rühlemann’s Kräuter und Duftpflanzen (Horstedt, Germany) and sown into plant pots with 1 L soil (Einheitserde classic, Einheitserde Werkverband e.V., Sinntal-Altengronau, Germany; pH = 5.9, N = 183 mg/L, P_2_O_5_ = 135 mg/L, K_2_O = 212 mg/L). The average temperature was 20 °C during the day and 18 °C at night in the climate chamber at the Leibniz Institute of Vegetable and Ornamental Crops (IGZ) e.V. The light intensity was set to 150 μmol/m^2^/s, the CO_2_ concentration to 380 ppm, and the humidity to 70% for both the screening experiment and the basil used for the preparation of the pesto.

Ten mature leaves were harvested after 6 weeks (plant height approximately 15 cm) from 5 different pots and directly frozen in liquid nitrogen. Prior to extraction, 0.5 g of basil leaves and 2 g of sodium chloride were homogenized in liquid nitrogen using a pestle and mortar. An aliquot of 25 mg was mixed with 10 mL of water/methanol (95:5, v/v) and taken for the extraction of volatiles by stir-bar-sorptive extraction for 20 min. Afterward, the stir bars were washed and stored in sealed vials until GC–MS analysis of ME and eugenol. To this end, the GC–MS system was operated in full-scan mode and quantification of both alkenylbenzenes was achieved via external calibration (as the internal standard, *d*_3_-ME, was not yet available). Detailed instrumental settings are given in the Supporting Information.

### Determination of the ME Content in Basil Leaves and Pesto by GC–MS/MS using an Isotopically Labeled Internal Standard

When *d*_3_-ME was available, we found that the recovery was incomplete with the method described in the preceding paragraph, which involved stir-bar-sorptive extraction combined with GC–MS analysis in the scan mode. Therefore, it was replaced in favor of a procedure utilizing organic solvents accompanied by the synthesized *d*_3_-ME as an internal standard for extraction and GC–MS/MS analysis in multiple reaction monitoring (MRM) mode. Prior to extraction with methanol-saturated hexane, 0.6 g of basil pesto (recipe see Suppl. Table S2) and 2 g of sodium chloride were homogenized in liquid nitrogen using a pestle and mortar. Aliquots of this homogenate (18 ± 2 mg) were weighed into glass vials with screw caps; 2 μL of a *d*_3_-ME solution in hexane (212 ng) was added each, and the samples were subsequently filled up to 1 mL with methanol-saturated hexane. These mixtures were stirred at 400 rpm at room temperature for 2 h. Afterward, the extracts were filtered through sodium sulfate. The resulting eluates were analyzed via GC–MS/MS (Suppl. Figure S5). The method was also applicable to basil leaves. Here, only 0.24 g of fresh basil leaves were used in the homogenization step.

The development of the GC–MS/MS analysis referred to a method described previously.^35^ The GC–MS system consisted of an Agilent 7890B GC-System (Agilent Technologies, Waldbronn, Germany) with a MultiPurpose Sampler (Gerstel, Mülheim an der Ruhr, Germany) coupled to an Agilent 7010 triple-quadrupole (QQQ) mass spectrometer (Agilent Technologies). For chromatographic separation, 1 μL was injected in splitless mode with a 20 mL/min purge flow to a split vent at 1.0 min. Helium with a constant flow rate of 1.2 mL/min was used as the carrier gas. The temperature for the inlet was 280 °C. Analyte separation was achieved on an HP-5MS column (0.25 mm × 30 m, 0.25 μm; Agilent Technologies) using the following temperature program: starting at 40 °C (held for 2 min), the oven temperature initially increased to 150 °C at a rate of 30 °C/min, to 210 °C at a rate of 5 °C/min, and, subsequently, to 325 °C (held for 2 min) at a rate of 50 °C/min. The solvent delay time was set to 4 min. Analytes were ionized in an electron ionization (EI) interface with an electron energy of 70 eV at a temperature of 230 °C. For the MRM mode, nitrogen at a flow rate of 1.5 mL/min was used as collision gas, whereas helium at a flow rate of 2.25 mL/min was used as quench gas. MRM transitions for ME and *d*_3_-ME were tuned manually resulting in the following fragmentation (collision energies in parentheses): *m*/*z* 178.1 → 163.0 (5 eV), *m*/*z* 178.1 → 147.0 (5 eV), *m*/*z* 178.1 → 107.0 (15 eV) for ME and *m*/*z* 181.1 → 166.0 (5 eV), *m*/*z* 181.1 → 150.0 (5 eV), *m*/*z* 181.1 → 107.0 (15 eV) for *d*_3_-ME. The fragmentation which yields the anisole fragment ion (*m*/*z* 178.1 → 107.0 and *m*/*z* 181.1 → 107.0) served as a quantifier (Suppl. Figure S4). For data acquisition and processing, MassHunter GC/MS Data Acquisition (Version B.07.02.1938) and MassHunter Qualitative Analysis (Version B.07.00) were used (Agilent Technologies).

### Exposure Study in Humans

To evaluate the formation and urinary excretion of MEMA in humans, a pilot investigation involving a single volunteer (the experimenter) was conducted. The experimenter consumed 91 g of basil leaves mixed with basil-flavored olive oil (the amount of ME ingested was not determined in this pretest). Thereafter, complete urine was collected in self-chosen intervals over a period of 24 h. Control urine was obtained prior to the consumption of the study meal. To ensure that the control urine was free of MEMA and no additional ME uptake occurred after the controlled exposure, the participant avoided ME-containing food 2 days before and during the urine sampling. The urine was collected in a measuring cup, its volume was noted, and a sample of ∼40 mL was stored at −20 °C until analysis.

Based on the findings of the pilot investigation, a more comprehensive (“main”) exposure study was performed. The study was conducted according to the guidelines of the Declaration of Helsinki and was approved by the ethics committee of the University of Potsdam under application number 4/2019. It was carried out at the Institute of Nutritional Science (University of Potsdam), where 12 healthy volunteers (six men and six women) with conventional nutrition habits were recruited. All participants gave written informed consent. The mean age of the test group was 31.6 ± 4.6 years (range: 25–42 years), and the mean body weight was 76.4 ± 10.0 kg (range: 61–94 kg). Characteristics of the cohort of participants are presented in Table 1. To guarantee that urinary excretion of MEMA was solely attributed to the consumption of the basil pesto served, all volunteers were asked to avoid ME-containing food 2 days before and after the consumption of the study meal. Therefore, they received an information sheet specifying potential ME sources. After overnight fasting, the participants consumed a meal comprising 100 g of self-made pesto containing 30 g of basil (equal to 1.7 mg of ME; recipe see Table S2) and 200 g of pasta. No further restrictions were exerted. To ensure proper urine sampling, the volunteers were encouraged to drink beverages as they preferred. Urine was collected quantitatively in capped polyethylene containers prior to the controlled exposure and during the following intervals: 0–1, 1–2, 2–3, 3–4, 4–5, 5–6, 6–8, 8–10, 10–12, 12–24, 24–36, and 36–48 h. After the volumes were measured, the urine was aliquoted in two 15 mL reaction tubes per time interval and stored at −20 °C until analysis.

**Table 1:** Basic Data of the Participants and Estimates of the Total Urinary *E*-3′-MEMA Excretion of 12 Participants following the Consumption of 100 g Basil Pesto Containing 1.7 mg of ME^a^

participant	age [y]	gender	body weight [kg]	smoker	*E*-3′-MEMA	
					ng	ratio of ME intake [ppm]
1	31	m	76	no	12	4
2	42	m	89	no	51	16
3	35	m	94	no	18	6
4	28	f	61	no	36	11
5	37	m	75	no	20	6
6	31	m	77	no	52	16
7	28	m	69	yes	105	33
8	31	f	61	no	63	20
9	30	f	80	no	274	85
10	28	f	85	no	44	14
11	33	f	77	yes	3	1
12	25	f	73	no	6	2

a The *E*-3′-MEMA amount (ng) is the total of excretion considering all urine samples with *E*-3′-MEMA concentrations greater than the LOD.

### Establishment of MEMA Extraction from Human Urine

For the establishment of an extraction protocol of MEMA from human urine as well as the development of LC–MS/MS analytics, the synthesized MEMA isomer mixture was used. First, the following organic solvents were selected to study extraction efficiency: *n*-butanol, methyl *tert*-butyl ether, *n*-butyl acetate, EtOAc, and isoamyl alcohol. Urine (pH 6.1) serving as a matrix for the extraction experiments was obtained from a volunteer after 1 week of abstinence from foods containing ME. To this end, 2 mL of urine, untreated or adjusted to pH 4 or pH 2 with HCl, was placed in a 15 mL tube. Then, 10 μL of 2.5 μM MEMA in methanol was added followed by vortexing and brief centrifugation. For MEMA extraction, 2 mL of the organic solvent was added. The mixture was vortexed vigorously for 1 min and phase separation was facilitated by centrifugation at 1500 × *g* for 10 min. The organic (upper) phase was transferred to a new 15 mL sample tube and evaporated to dryness using a Savant SpeedVac concentrator (Thermo Fisher Scientific, Dreieich, Germany). In the case of doubled extraction, the aqueous layer was repeatedly extracted as stated above and both organic phases obtained were combined before vacuum-assisted drying. The dry residue was taken up in 1 mL of methanol, vortexed vigorously for 1 min, and ultrasonicated for 10 min. The mixture was quantitatively transferred to a 1.5 mL Eppendorf tube and again concentrated to dryness in the SpeedVac. The final residue was taken up in 50 μL of methanol followed by vigorous vortexing for 1 min and ultrasonication for 10 min. After centrifugation at 16,000 × *g* for 10 min, the supernatant was subjected to LC–MS/MS analysis. Matrix-matched external calibration was performed to determine recovery rates and evaluate the matrix effects. For this purpose, urine was processed without MEMA spiking, as described above. Final dry residues were then dissolved in 50 μL of calibration solutions (50, 100, 250, 500, and 750 nM MEMA in methanol).

As an alternative approach, solid-phase extraction (SPE) methods were conducted. Hence, three different SPE cartridges were tested: Chromabond C18 (3 mL/500 mg), Chromabond C18 end-capped (C18ec, 3 mL/500 mg) (both from Macherey-Nagel, Düren, Germany), and Isolute ENV+ (3 mL/100 mg) from Biotage (Uppsala, Sweden). Urine (2 mL) of the volunteer was filled to 4 mL with 50 mM ammonium formate buffer, adjusted to pH 2.5 with formic acid, and spiked with 10 μL of 2.5 μM MEMA in methanol. Solid phases were conditioned with 4 mL of methanol, 2 mL of water, and 2 mL of 0.1% formic acid (pH 2.5). Vortexed and centrifuged samples were applied and allowed to pass through the cartridges before the sorbents were washed with 0.5 mL of 0.1% formic acid (pH 2.5) and 0.4 mL of 0.1% formic acid (pH 2.5)/methanol (9:1, v/v). After drying the solid phases, retained analytes were eluted once or twice by three consecutive applications of 0.5 mL of 1% formic acid in methanol. The eluates were concentrated to dryness using the SpeedVac and the dry residues were taken up in 50 μL of methanol or methanolic MEMA standards in order to create matrix-matched calibrants. The further procedure here was the same as that for liquid–liquid extraction described above. All extraction methods investigated, whether using organic solvents or solid phases, were evaluated in terms of MEMA recovery and detection sensitivity influenced by coextracted matrix components. The latter was assessed by the slopes of the linear calibration functions.

### Analysis of MEMA by LC–MS/MS

Two different instrumental LC–MS/MS setups were used for the development of the methodology on the one hand and the final analytics of the exposure study in humans on the other hand. In the following, the setup for method development and initial analyses of urine from a single volunteer is referred to as “system 1” and that for the analysis of the main exposure study including 12 subjects as “system 2”.

“System 1” consists of the following instrumentation: 1260 Infinity HPLC coupled via an AJS electrospray ionization (ESI) interface to a 6490 QQQ mass spectrometer or a 6530 quadrupole-time-of-flight (QTOF) mass spectrometer (all Agilent Technologies). The following chromatographic conditions were found to be optimal for the separation of MEMA isomers present in the synthesized reference material with good sensitivity. Five microliters of urine extract were injected into a mobile phase system consisting of 96% 1 mM ammonium acetate (eluent A) and 4% acetonitrile (eluent B). The mobile phase was pumped with a constant flow of 0.5 mL/min and the following gradient elution program was used (proportion of eluent B is given): 0 → 1 min: 4%; 1 → 2.5 min: 4 → 20%; 2.5 → 11.5 min: 20 → 40%; 11.5 → 13.5 min: 40 → 80%; 13.5 → 15 min: 80%; 15 → 15.01 min: 80 → 4%; 15.01 → 18 min: 4%. Separation occurred within a Poroshell 120 EC-C18 column (3.0 × 150 mm, 2.7 μm) guarded by a precolumn (4.6 × 5 mm, 2.7 μm) of identical material (Agilent Technologies). During analysis, samples were cooled to 4 °C and the column was kept at 30 °C. Regarding polarity of electrospray ionization, the negative mode (ESI-) proved to be advantageous and was therefore applied for further measurements. To establish a MRM method, mass transitions were optimized in an automated manner by using the *Optimizer* tool (version B.06.00) of the MassHunter software (Agilent Technologies) for the QQQ instrument. The following MRM transitions were obtained (collision energies in parentheses): *m*/*z* 338.1 → 208.9 (12 eV), *m*/*z* 338.1 → 193.9 (24 eV), and *m*/*z* 338.1 → 178.8 (36 eV). The loss of the sulfur-free NAC moiety (*m*/*z* 338.1 → 208.9) served as a quantifier (Figure 1). Using the developed MRM method, the ion source parameters were then optimized using the QQQ mass spectrometer and the *Source and iFunnel Optimizer* (version B.06.00) software tool (Agilent Technologies): sheath gas temperature, 400 °C; sheath gas flow, 12 L/min of nitrogen; nebulizer pressure, 20 psi; drying gas temperature, 280 °C; drying gas flow, 11 L/min of nitrogen; capillary voltage, 4000 V; nozzle voltage, 500 V; iFunnel high pressure RF voltage, 130 V; and iFunnel low pressure RF voltage, 120 V. For accurate mass measurements, the QTOF mass spectrometer was used. The parameters optimized for the HPLC-QQQ system were adopted as far as possible.

“System 2” was configured as follows: an HPLC 1100 (Agilent Technologies) was connected to a QTrap6500 triple quadrupole-hybrid ion trap mass spectrometer (Sciex, Darmstadt, Germany) equipped with an electrospray ionization source operating in negative mode. The chromatographic separation of the analytes was performed by ion pair chromatography on a Nucleoshell RP 18plus column (2.0 × 150 mm, 2.7 μm; Macherey-Nagel). The eluents were water containing 10 mM tributylamine and 10 mM acetic acid (eluent A) and acetonitrile (eluent B). The flow rate of the gradient (0 → 1 min: 2% eluent B; 1 → 8 min: 2 → 15% eluent B; 8 → 17.5 min: 15 → 35% eluent B; 17.5 → 18 min: 35 → 100% eluent B; 18 → 20 min: 100% eluent B; 20 → 20.1 min: 100 → 2% eluent B; 20.1 → 23 min: 2% eluent B) was 0.5 mL/min. The temperature of the column oven was set to 40 °C and the sample injection volume was 5 μL. The operating parameters of the QTrap6500 were ion spray voltage, −4500 V; interface heater temperature, 450 °C; curtain gas, 40 psi; ion source gas 1, 60 psi; ion source gas 2, 50 psi; collision activated dissociation gas set to medium. The MRM mode was employed for quantitative analysis with the declustering potential and the entrance potential at −30 and −10 V, respectively. For the quantifier mass transitions of *E*-3′-MEMA (*m*/*z* 338.1 → 209.1) and *d*_6_-*E*-3′-MEMA (*m*/*z* 344.1 → 215.1), a collision energy of −20 V and a cell exit potential of −5 V were used. Data acquisition and processing were carried out using Analyst version 1.7.1 software (Sciex).

### Method Validation

The comprehensive validation of the *E*-3′-MEMA detection was only performed for “system 2″, as this configuration and approach was used for *E*-3′-MEMA quantification in the main exposure study. The linearity of detection, matrix effect, LOD and LOQ were determined by a dilution series of *d*_6_-*E*-3′-MEMA in water or urine (pool of 5 subjects). The effect of the sample matrix on the mass spectrometric detection of the analytes was assessed by comparing the slopes of the calibration line of *d*_6_-*E*-3′-MEMA determined in water with those determined in the presence of extracts of pooled urine samples (matrix). The LOD (signal-to-noise ratio (S/N) = 3) and LOQ (S/N = 10) were determined from the calibration line of *d*_6_-*E*-3′-MEMA prepared with or without the urinary matrix. The intraday and interday precision of the method was determined by analyzing urine samples (pool of spot urine samples from 5 subjects) spiked with three different concentrations of MEMA as described above (20, 200, and 1000 nM; intraday precision = 6 replicates; interday precision = 5 replicates).

### Data Analysis

The *E*-3′-MEMA concentrations in 64 out of 156 urine samples were below the LOD. These data were substituted with LOD/2. The values above the LOD but lower than the LOQ (*n* = 83) were used as such, as a higher validity of results can be expected compared to setting all these values to half of the LOQ.^36^

Data analyses were conducted with SigmaPlot version 14.0 (Systat Software, Inc., Erkrath, Germany). The hourly urinary *E*-3′-MEMA excretion levels of the 12 study participants were presented as median values and interquartile ranges.

## Results

### Selection of a Basil Cultivar and GC–MS/MS Quantification of ME in Basil Pesto Utilized for Controlled Exposure

In an initial screening experiment, 18 different basil cultivars were grown under controlled conditions and their contents of ME as well as its biosynthetic precursor eugenol were determined by GC–MS. It turned out that the cultivars differed greatly in the amount of these two alkenylbenzenes (Suppl. Table S1). Except for one cultivar, the eugenol content exceeded that of ME (up to 408-fold). Half of the basil cultivars investigated had levels <10 μg ME/g fresh weight. Only three cultivars had ME levels >100 μg/g, with “Genoveser” basil showing the highest ME content (138.2 μg/g). Therefore, this cultivar was chosen for the production of the basil pesto to be served in the main exposure study.

Plants were cultivated in a climate chamber under defined conditions to immediately produce the pesto (recipe see Table S2) after harvest. Afterward, samples were taken and analyzed by the established isotope-dilution GC–MS/MS method (Supporting Information, Figure S5). As a result, ME contents of 47 and 17 μg/g were determined in basil leaves and pesto, respectively. The basil pesto was stored at −80 °C until the day of exposure and then thawed at room temperature.

### LC–MS/MS Detection of MEMA

The purified product of the MEMA synthesis was first characterized by using high-resolution mass spectrometry (HRMS). An accurate mass of *m*/*z* 338.1069 was detected, which, compared to the theoretical mass (C_16_H_21_NO_5_S, [M – H]^−^*m*/*z* 338.1068), proves the molecular composition of the reference material with mass inaccuracy of less than 1 ppm. A coupling of HPLC and HRMS resulted in signal splitting in the single ion monitoring (SIM) chromatogram to three prominent peaks (and a tiny additional peak) after the optimization of chromatographic conditions (Figure 1A). Each of the three prominent signals had a molecular ion with identical accurate *m*/*z* ratio in the mass spectrum (Figure 1B), indicating that they must be isomeric compounds. Collision-induced dissociation (CID) of the precursor ion (*m*/*z* 338.1) yielded at least three characteristic product ions (Figure 1C and Suppl. Figure S1), which can be assigned to specific fragmentations in the molecule (Figure 1D) and were subsequently used to establish an MRM method (Figure 1E). The stable-isotope-labeled reference material, *d*_6_-MEMA, was characterized in an analogous manner by LC–MS/MS (Suppl. Figure S3). HRMS confirmed its identity by determining the accurate mass (*m*/*z* 344.1467) with a mass error of less than 10 ppm (C_16_H_15_D_6_NO_5_S, [M – H]^−^*m*/*z* 344.1444). Again, the chromatographic signal was split into three prominent peaks (and a tiny additional peak), all originating from molecular ions of identical *m*/*z* ratio. After CID, product ions were obtained that followed the same fragmentation pattern as that of the unlabeled analogue. In a study on the excretion of mercapturic acids of estragole after consumption of fennel tea, we recently observed a similar chromatographic behavior–signal splitting into at least three isomers.^37^ In that study, regioselective synthesis identified the main signal, third in the elution order, as the *E*-3′-conjugated isomer. Therefore, regioselective synthesis of the *E*-3′-isomer was also targeted for MEMA. The obtained isomerically pure product, *N*-acetyl-*S*-[3′-(3,4-dimethoxyphenyl)allyl]-l-cysteine (*E*-3′-MEMA), verified the identity of the MEMA main signal (Peak 3, see Figure 1A). The identities of Peaks 1 and 2 of the chromatographic analysis of MEMA (Figure 1A) remains elusive. We speculate that these are a pair of diastereomers of the 1′-conjugation of NAC and ME. A hypothesis that would need to be verified in further studies using NMR spectroscopy. The *E*-3′-MEMA standard was then used to quantify the main signal (Peak 3) of the *d*_6_-MEMA isomer mixture (Suppl. Figure S3A), which could thus be used for isotope-dilution LC–MS/MS analysis of *E*-3′-MEMA in urine samples collected after exposure to ME-containing basil pesto in humans.

### Extraction of MEMA from Human Urine

Next, the extraction of MEMA from the spiked urine was optimized. Of the solvents tested (*n*-butanol, methyl *tert*-butyl ether, *n*-butyl acetate, EtOAc, and isoamyl alcohol), only *n*-butanol resulted in satisfactory recoveries (45–54% for the three MEMA isomers) in urine that was not pH-adjusted (usually ∼ pH 6.1). Double versus single extraction did not significantly improve recoveries. Subsequently, the influence of a pH decrease on the extractability of MEMA was tested. Spiked urine, adjusted to pH 2 or pH 4, was extracted with *n*-butanol or EtOAc. This modification resulted in a remarkable increase in recovery compared to nonadjusted urine. For the three MEMA isomers, these were 81–89 and 70–82% for pH 2 and 4, respectively, for *n*-butanol. For EtOAc, these were 54–84% (pH 2) and 23–68% (pH 4). A double extraction tended to reduce rather than increase the recovery rates, probably due to increased carry-over of signal-quenching matrix components. SPE has led to even higher recoveries. When eluted twice, these were 82–109%, 77–115%, and 65–96% for the Isolute ENV+, Chromabond C18, and Chromabond C18ec cartridges, respectively. However, in addition to the recoveries, the matrix effects, expressed by the slopes of the matrix-matched MEMA calibration functions, also contributed to the decision for the extraction method. The slopes *m* of the linear calibration (*y* = *mx* + *n*) for the *E*-3′-conjugate of MEMA, were at least 4-fold higher for extraction with EtOAc than those for *n*-butanol or the SPE columns tested. The slopes *m* (in 1/nM) accounted for 82.4 (EtOAc, pH 2), 182.7 (EtOAc, pH 4), 19.3 (*n*-butanol, pH 2), 24.3 (*n*-butanol, pH 4), 18.0 (Isolute ENV+), 18.7 (Chromabond C18), and 22.6 (Chromabond C18ec).

In the end, we used and recommended the following extraction protocol for the determination of MEMA in human urine: after thawing and vortexing of the sample, 4 mL of urine is transferred into a 5 mL reaction tube. For acidification of the urine to pH 2, 25 μL of aqueous HCl (32%) along with 50 μL of *d*_6_-*E*-3′-MEMA (133 nM in methanol) as isotope-labeled internal standard are added to each sample followed by vortexing for 1 min. After centrifugation (5 min, 3500 × *g*, room temperature), two 1.5 mL aliquots of the supernatant are transferred to new 5 mL reaction tubes, and 1.5 mL of EtOAc are added to each of them. Thereafter, the samples are vortexed vigorously for 1 min and centrifuged (5 min, 3500 × *g*, room temperature). The organic phase of the samples is transferred to a 2 mL reaction tube and stored at −80 °C for 60 min. The solvents are evaporated by vacuum centrifugation at 10 mbar. After 25 min of drying, the two extracts are combined and evaporated to dryness. The dried samples are reconstituted in 50 μL of methanol, centrifuged (5 min, 12,000 x *g*), and transferred to HPLC vials.

### Validation of the LC–MS/MS Method Applied for Quantification of *E*-3′-MEMA in Human Urine

LOD, LOQ, and the linearity of detection of the established LC–MS/MS method (setup “system 2”) were determined by a serial dilution of *d*_6_-*E*-3′-MEMA in pure water or urine samples (pool of 5 subjects) that were processed by liquid–liquid extraction. In both cases, linear regression of the MS signal intensities over the tested concentration range between 0.25 and 500 nM yielded coefficients of determination (*R*^2^) of >0.99 (Suppl. Figure S6). In samples with the urine matrix, a signal reduction (matrix effect) of 46.1% was observed. The LOD (S/N = 3) and LOQ (S/N = 10) values without matrix were 2.5 and 7.5 fmol on column, respectively, whereas with matrix, LOD and LOQ values were 50 and 150 fmol on column, respectively. However, it is of note that the LOD and LOQ depended much on the intensities of the individual background signals showing a significant variation among the human samples. Thus, a sample-specific LOD was defined as three times the intensity of the noise at the retention time of the *E*-3′-MEMA signal. The LOQ was defined as three times the LOD. No carry-over was detected in the analyzed concentration range. For the determination of the intraday (*n* = 6 replicates) and interday precision (*n* = 5 replicates) of the final method, four concentrations of *E*-3′-MEMA (0, 20, 200, and 1000 nM) were spiked to a urine sample (pool of 5 subjects) and processed by liquid–liquid-extraction. The intraday precision ranged from 4.3 to 9.6% (CV) whereas the interday precision ranged from 5.6 to 18.3% (CV).

### Detection of MEMA Isomers in the Urine of a Volunteer (Pilot Investigation)

**Figure 2:**
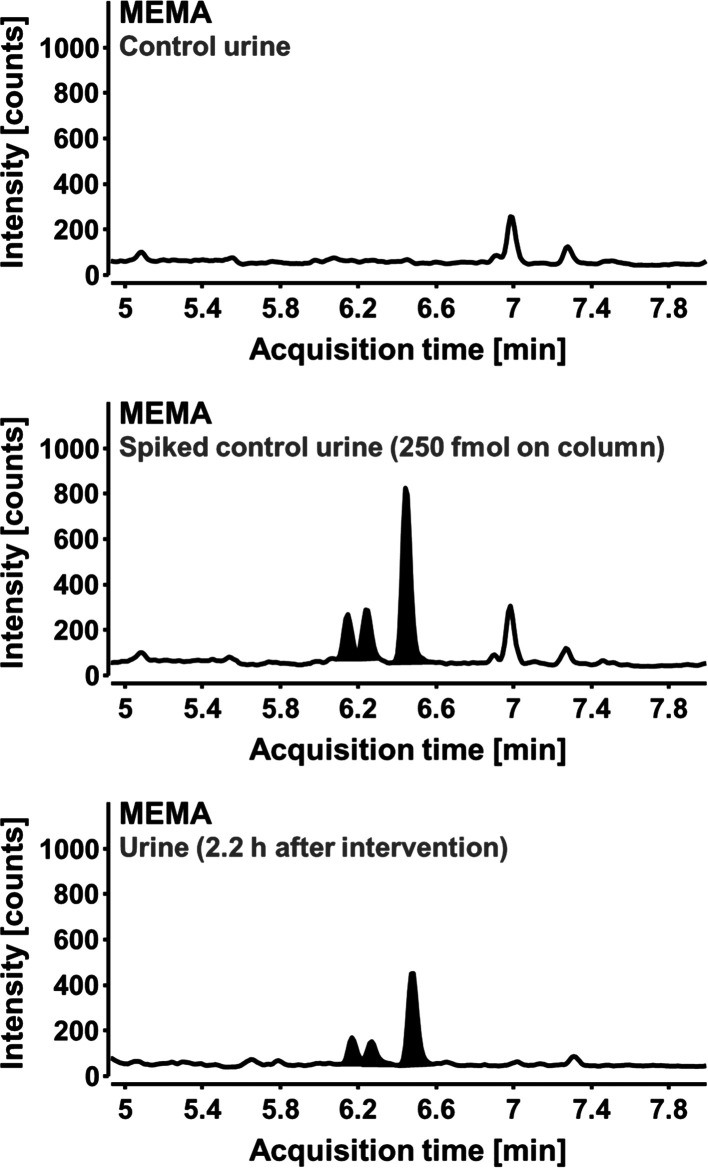
Proof of MEMA isomers in human urine using LC–MS/MS setup “system 1”. MEMA was detected in urine of a volunteer after consumption of ME-containing food (91 g of basil mixed with basil-flavored olive oil). Urine was collected over a period of 24 h. MEMA excretion was time-dependent. The highest signal intensities were obtained in urine collected 2.2 h after consumption of basil leaves and are shown in the lowest chromatogram. MEMA signals were absent in control urine (top chromatogram) and coeluted with those of a spiked urine sample (middle chromatogram). Chromatograms were recorded in ESI- MRM mode, but only the quantifier mass transition (*m*/*z* 338.1 → 208.9) is shown. All signal peaks (shaded) also exhibited both qualifier MRM transitions (*m*/*z* 338.1 → 193.9 and *m*/*z* 338.1 → 178.8).

With optimized extraction and MRM analysis methods at hand, a pilot investigation was conducted on the urine of a volunteer who consumed ME-containing food on a single occasion. As shown in Figure 2, the control urine collected before the meal was free of MEMA signals in the quantifier mass transition. However, in urine collected 2.2 h after the consumption, three isomeric peaks for MEMA were detected, which coeluted with the signals from a spiked control urine sample. Interestingly, the intensity ratios of the isomers were almost identical in the chemically synthesized material as well as in the metabolized product, which herewith could be detected for the very first time in human urine. This finding prompted us to initiate a more comprehensive exposure study to investigate the kinetics of MEMA excretion.

### Level and Time Course of Urinary Excretion of *E*-3′-MEMA in Participants of the Exposure Study

**Figure 3:**
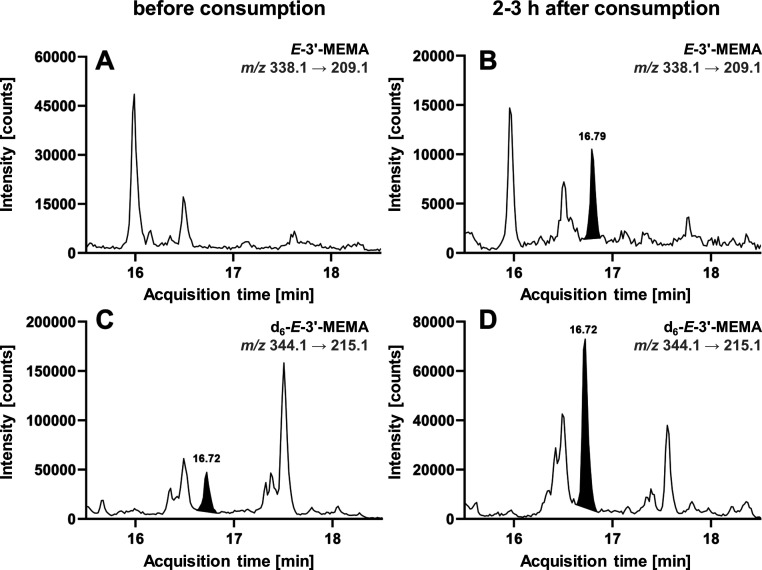
LC–MS/MS detection of *E*-3′-MEMA in human urine collected before (A,C) and 2–3 h after the consumption of ME-containing basil pesto (B,D). Shown are the quantifier mass transitions of *E*-3′-MEMA (A,B; *m*/*z* 338.1 → 209.1) and *d*_6_-*E*-3′-MEMA (C,D; *m*/*z* 344.1 → 215.1). Corresponding signals of *E*-3′-MEMA and its internal standard are shaded black. LC–MS instrumental setup “system 2” was applied.

In the exposure study, urinary *E*-3′-MEMA of the 12 participants was analyzed before (one sample) and in the first 48 h (12 samples) after the consumption of the basil pesto meal containing 1.7 mg ME. Exemplary MRM chromatograms of samples of one participant collected before and 2–3 h after the consumption are shown in Figure 3. In the urine samples collected before the exposure, no *E*-3′-MEMA was detected (except for a tiny peak in the range of the LOD in two subjects). This can also be seen in Figure 3A. At the retention time of the deuterated internal standard *d*_6_-*E*-3′-MEMA (16.72 min), no signal could be detected in the mass transition for *E*-3′-MEMA. However, this changed in the samples collected within the first 3 h after the ME-containing meal. Here, clear signals for *E*-3′-MEMA were observed at the retention time of interest (Figure 3B).

**Figure 4:**
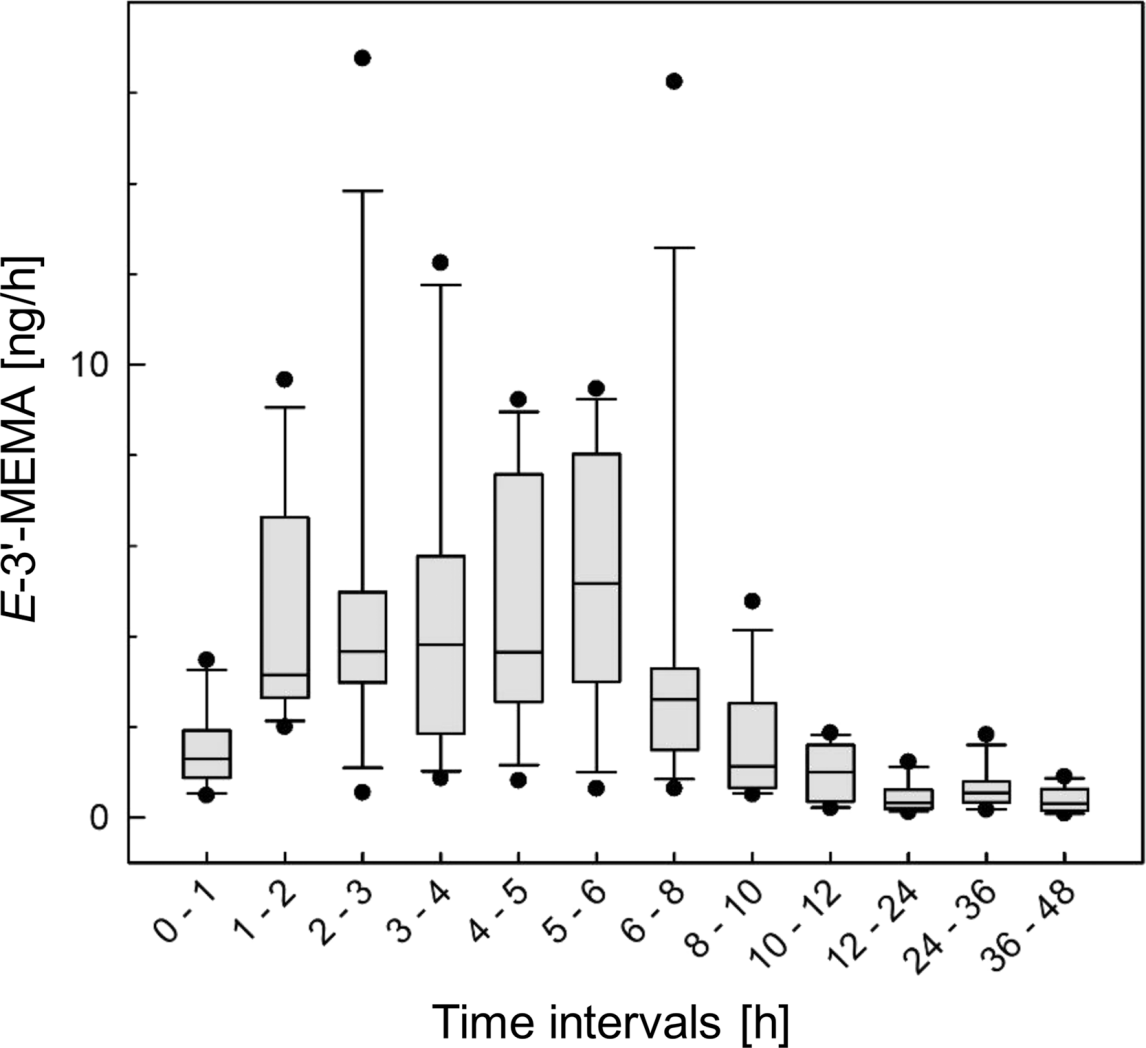
*E*-3′-MEMA excretion in the urine samples collected from 12 subjects after the consumption of ME-containing basil pesto. Lines and boxes represent median values and the lower and upper quartiles, respectively. The bars represent the 10th and 90th percentiles.

The concentrations of *E*-3′-MEMA were analyzed in all urine samples, and the hourly excretion rates were calculated for each time interval, except for the spot urine collected before the consumption. After exposure to the ME-containing basil pesto, an instantaneous increase in the urinary excretion rates of *E*-3′-MEMA was observed (Figure 4). The highest individual excretion rates of *E*-3′-MEMA were reached in the time intervals between 1 and 2 and 5–6 h after consumption. After 12 h, *E*-3′-MEMA was usually not detectable in the samples collected later (24, 36, and 48 h after the consumption). The overall excretion was estimated for all study participants by summing up the amounts of *E*-3′-MEMA in the samples with detectable levels (Table 1, between 3 and 274 ng, corresponding to 1 and 85 ppm of the ME intake), after subtraction of a mean background concentration. This was estimated for each participant as the mean theoretical *E*-3′-MEMA concentration in all samples with signals below the LOD (which were set to LOD/2). This approach allowed conservative estimates for the urinary excretion of *E*-3′-MEMA in individual participants.

## Discussion

### Concept and Pilot Investigation

ME is a genotoxic carcinogen in rodents.^3^ DNA adducts, found in rodents after ME treatment,^12^ have also been detected in human liver^16,17^ and lung samples.^18^ Apart from that, no additional products of the metabolic activation of ME in humans have been reported so far. Thus, there is a need for noninvasive biomarkers to obtain information about the exposure, relevant for the risk assessment of ME in humans.

In the current study, we aimed to identify mercapturic acids of ME in the urine of volunteers after consumption of a typical ME-containing meal. MEMA is a detoxification product resulting from the conjugation of metabolically activated ME with GSH and subsequent enzymatic conversion (Scheme 1). This reaction may occur spontaneously or be mediated by GSTs. Some reactive sulfo conjugates, such as 1-menaphthyl sulfate (1-hydroxymethylnaphthalene) and 5-hydroxymethylchrysene, are substrates for rat GST T1 and T2 (previously termed GST Yrs’ and Yrs).^38^ It is not known whether 1′-sulfooxy-ME is a substrate for these or any other GST forms. Additionally, mercapturic acids may be formed from the reaction of the electrophilic metabolite with free cysteine (which, however, is much less abundant in cells than GSH) and cysteine residues in proteins (followed by protein degradation, a process usually requiring much time). Mercapturic acids are usually excreted into the urine, making these metabolites possible noninvasive biomarkers for exposure to the reactive intermediate.^39,40^ By employing a chemically synthesized MEMA standard, consisting of at least three isomers (Figure 1), we were able to establish an LC–MS/MS method for the determination of MEMA in human urine. A pilot investigation pointed out that MEMA was excreted in urine within about 12 h after ME intake (as basil leaves) and, interestingly, in a similar pattern compared to the isomer mixture of the MEMA standard (Figure 2). This outcome would be plausible with a purely chemical reaction of the electrophilic ME intermediate with cysteine residues, whereas product selectivity might be expected for enzyme (GST)-mediated conjugation.

### ME Content of Basil used in the Main Exposure Study

Further investigations were conducted within a controlled exposure study with 12 participants. In this context, basil pesto was chosen as ME-containing meal because it is a popular food and is thought to lead to high intake levels of ME.^41^

In preparation, 18 different basil cultivars were analyzed for their ME content (Suppl. Table S1). The basil cultivars varied greatly in the content of ME detected, underlining the importance of this prior investigation. Thereby, no clear connection between the contents of eugenol, a precursor in the synthesis of ME,^42,43^ and ME was seen. “Genoveser” basil was chosen to produce the pesto for the exposure study, as it showed the highest ME content. However, analysis of the self-made pesto revealed a total content of 17 μg ME/g pesto only, which is in the range of values known from the literature (0.01–99.3 μg ME/g pesto^44−47^). The substantial difference between the ME content of “Genoveser” basil initially found (Suppl. Table S1) and the comparatively low level in the self-made basil pesto may be ascribed to several possible factors: (a) The analytical methods used for ME determination in the basil cultivars and in the pesto for the exposure study differed. Specificity and correction for incomplete recovery were improved in the pesto analysis, involving an isotopically labeled internal standard and MS/MS methods. (b) Although the same “Genoveser” cultivar for the production of the basil pesto was planted, ME content can differ within each plant batch, as the ME content of basil is affected by various factors, e.g., developmental stage,^48^ size of the leaves,^42^ and drought^43^ or cold stress.^49^ Although we cultivated the plants in a climate chamber under defined conditions, standardization was not sufficient. Notwithstanding that the ME amount in our self-made pesto was not outstandingly high, it was assumed to be sufficient for an exposure study in humans.

### Findings in the Main Exposure Study

Excretion of *E*-3′-MEMA occurred soon after consumption of the basil pesto and ended after 12 h besides minor signals (close to the LOD) in three participants. In this respect, the excretion behavior of *E*-3′-MEMA was similar in all participants. Major differences were observed regarding the total amount (3–274 ng/person corresponding to 1–85 ppm of the ME intake, Table 1), which is comparable to the excretion of the mercapturic acid of estragole (13.2 ppm, *n* = 1).^37^ In addition, the peak urine elimination of *E*-3′-MEMA varied between 1 and 2 and 5–6 h, implying that the elimination of ME was rather fast in all study subjects, in agreement with the findings of human toxicokinetic studies conducted by Schecter et al.^50^ In that study, ME-rich gingersnaps were given to 12 volunteers. Serum levels of ME were determined before the meal and 5–120 min after the meal. Peak levels were observed 5 min after the meal, and the half-life of elimination was about 90 min.

Thus, the principal findings of our exposure study are as follows: (1) *E*-3′-MEMA was excreted in urine, (2) its levels were very low, and (3) they substantially varied among the study participants.

### Possible Reasons for the Low Urinary Excretion of *E*-3′-MEMA

The formation of *E*-3′-MEMA requires several sequential metabolic steps: 1′-hydroxylation, sulfonation, GSH conjugation, and processing of the GSH conjugate; competing pathways may occur on each level.

1′-Hydroxylation represents the dominating metabolic pathway of ME in hepatic microsomal systems from various species, including humans, at high substrate concentrations (as outlined in the introduction). Likewise, the ratio of the levels of DNA adducts formed in mouse liver by equimolar doses of ME and 1′-hydroxy-ME was 1:2 in wild-type mice (and 1:3 in mice with human SULT1A1–1A2 transgenes),^12^ suggesting a similar conversion rate of ME into 1′-hydroxy-ME in these experimental models. However, the relative contribution of 1′-hydroxylation was reduced at low substrate concentrations in human liver microsomes at the expense of the 2′,3′-epoxidation. Al-Subeihi et al.^51^ determined the kinetic parameters for various human CYPs and oxidation reactions of ME and extrapolated them to the in vivo situation, taking into account the CYP levels present in the human liver. From the catalytic efficiencies of this model, we calculated a 38.4% contribution of the 1′-hydroxylation to the hepatic ME metabolism at low substrate concentration, somewhat less than that of the 2′,3′-epoxidation (58.8%).

The next step to be considered is the sulfonation of 1′-hydroxy-ME. The extent of the conversion of 1′-hydroxy-ME to 1′-sulfooxy-ME is difficult to estimate, since 1′-sulfooxy-ME is short-lived and not available as a chemical standard. Al-Subeihi et al.^26^ incubated 1′-hydroxy-ME with 3′-phosphoadenosine-5′-phosphosulfate (cofactor for SULT) and GSH and measured the amount of GSH conjugate formed. The rate (and the catalytic efficiency) of this pathway was extremely low in rat and human liver cytosolic fractions, lower by orders of magnitude than those of competing pathways, glucuronidation, and oxidation (to 1′-oxo-ME). This finding implies that either the sulfonation of 1′-hydroxy-ME to 1′-sulfooxy-ME, or the GSH conjugation of 1′-sulfooxy-ME, or both reactions were very slow. A competing reaction, the oxidation of 1′-hydroxy-ME to 1′-oxo-ME, is reversible.^26^ Alternatively, 1′-oxo-ME may undergo addition reactions at the 2′,3′-double bond with GSH^51^ – a type of conjugation reaction that often is reversible. The catalytic efficiency of 1′-hydroxy-ME glucuronidation, another competing reaction, was 125 times lower in liver microsomes from humans, as compared to male rats.^26^ Nevertheless, in rats treated with high doses of ME only, traces of the 1′-hydroxy-ME-glucuronide (detected as 1′-hydroxy-ME after glucuronidase/sulfatase treatment) were found in the urine.^27^ Interestingly, it appeared that 1′-hydroxy-ME-glucuronide was a major metabolite in bile (not accurately quantified). Nevertheless, since 95% of the dose of ME was recovered in urine as metabolites not formed via the 1′-hydroxy-ME-glucuronide, only a few percent of the dose of ME was excreted 1′-hydroxy-ME-glucuronide in bile at most. There were no indications for the presence of urinary or biliary metabolites formed via 1′-oxo-ME. As outlined in the Introduction, 1′-hydroxylation is the predominant initial pathway in liver microsomes from male rats (∼50% of the sum of all metabolites) at high ME concentrations (reflecting the situation of the metabolism study in rats, as the ME dose was high, 200 mg/kg). Therefore, the question arises what happened to the 1′-hydroxy-ME formed in vivo? In this context, it is interesting to note that 65% of the urinary metabolites (corresponding to 62% of the dose) appeared to be formed via 3′-hydroxy-MIE, although 3′-hydroxy-MIE plus 3′-oxo-MIE only contributed 10.3–47.6% to the metabolism of ME in liver microsomes from male rats (see introduction). Hydrolysis of 1′-sulfooxysafrole to 3′-sulfooxyisosafrole results in the formation of 1′-hydroxysafrole as well 3′-hydroxyisosafrole, probably via cleavage of sulfate resulting in the formation of the resonance-stabilized cation, which reacts with water; thus, SULTs may act as isomerases for 1′-hydroxysafrole as well 3′-hydroxyisosafrole.^52^ It has to be expected that the same SULT-mediated isomerization reactions occur with 1′-hydroxy-ME and 3′-hydroxy-MIE. These observations may suggest that a significant amount of the dose of ME (e.g., 14.3–41.7% on the basis of the numbers given above) was converted via 1′-sulfooxy-ME to 3′-hydroxy-MIE.

However, other observations tend to argue against a very extensive conversion of 1′-hydroxy-ME to 1′-sulfooxy-ME in animal models and humans in vivo. Thus, 1′-hydroxy-ME formed nine times higher DNA adduct levels in mice carrying a human SULT1A1–1A2 transgene compared to wild-type mice,^12^ implying that ≤1/9 of 1′-hydroxy-ME was converted to 1′-sulfooxy-ME in wild-type mice. Although human SULT1A1 was about 3 times more efficient in the activation of 1′-hydroxy-ME to a mutagen than its orthologue from the mouse,^10^ the SULT step is also limiting in the activation of ME in humans, as demonstrated by the association of ME DNA adduct levels with a copy number polymorphism, which in turn affected the levels of SULT1A1 mRNA and protein expression.^17^

The data from the present work and previous studies by other researchers^26,32,53^ imply that 1′-sulfooxy-ME can be converted to GSH conjugates. It is unknown whether this reaction occurs spontaneously or is mediated by enzymes. Furthermore, our results imply that further processing to mercapturic acids takes place in humans. GSH conjugates are preferentially converted to mercapturic acids and excreted in urine in humans, but the extent of conversion may vary among the different GSH conjugates. Notably, GSH and cysteine conjugates formed via the ME cation have been detected in bile and urine, respectively, of rats treated with ME. Thus, incomplete processing of the GSH conjugate and/or biliary excretion may have negatively affected the urinary levels of *E*-3′-MEMA detected in our study.

### Possible Reasons for the High Variation of *E*-3′-MEMA Excretion in Urine

Many different enzymes and transporters may be involved in the disposition of ME. The role of several individual human enzyme forms has been investigated. Thus, 1′-hydroxylation of ME at low substrate concentrations is conducted by several different CYPs, with a dominant role of CYP1A2, followed by CYP2C9.^51^ The major competing reaction is CYP2B6-mediated 2′,3′-epoxidation. The level of these enzymes is highly variable in human liver microsomes; thus, CYP1A2, 2B6, and 2C9 activities varied 117, 126, and 46-fold, respectively, in hepatic microsomes from 100 subjects.^54^

Four human SULTs [1A1, 1A2, 1C2 (termed 1C4 in a newer nomenclature), and 1E1], expressed in *Salmonella typhimurium*, were able to activate 1′-hydroxy-ME (each enantiomer) to a mutagen;^10^ taking into account the strength of the mutagenic effects as well as the protein expression of the SULTs in the human liver^55^ and the *Salmonella typhimurium* strains used,^56^ SULT1A1 clearly dominates the activation. SULT1A1 activity (using 4-nitrophenol as the substrate) in hepatic liver samples from 100 adult subjects varied by a factor of 5.3, calculated by dividing the 95th percentile by the fifth percentile.^57^ In another study, the highest and lowest SULT1A1 expression levels differed by a factor of 5.6 for mRNA and 4.5 for protein in a total of 121 liver biopsy samples.^17^ Others found that SULT1A1 activity in liver samples correlates nearly linearly with the number of SULT1A1 gene copies (1–5).^58^

1′-Hydroxy-ME is efficiently glucuronidated in hepatic microsomes from male rats;^26^ in humans, the efficiency of this pathway was lower by a factor of 125.^26^ Out of 12 recombinant UGT forms studied, only UGT 1A9 and 2B7 showed activity with 1′-hydroxy-ME as the substrate.^51^ Oxidation to oxo-ME, followed by GSH conjugation,^26^ is a further metabolic pathway of 1′-hydroxy-ME, competing with its toxification via sulfonation (with the reservation that these reactions may be reversible). The enzymes involved in this pathway have not been identified at a molecular level.

It is known that 1′-sulfooxy-ME is able to form GSH conjugates,^26,32,53^ but it has not been examined whether enzymes are involved in this reaction. If this were the case, human GSTT1 and T2 would be the primary candidates, as orthologous enzymes from the rat efficiently catalyzed the GSH conjugation of other electrophilically reactive sulfo conjugates.^38^ GSTT1 is missing in many subjects (38% of Caucasians) due to a deletion mutation.^59^

We have no information about interindividual variation in the processing of GSH conjugates to mercapturic acids apart from the textbook knowledge that serum γ-glutamyl transpeptidase in blood serum is elevated in hepatic diseases.

In a previous study, the levels of ME DNA adducts were investigated in liver samples of 121 Caucasians undergoing liver surgery.^17^ No information was available on the levels and time courses of the intake of ME. However, the mRNA levels of 323 pharmacogenes had been determined in the samples.^60^ A strong correlation was observed between adducts and SULT1A1 mRNA and ME DNA adduct levels (*p* = 1.1 × 10^–6^). Subsequently, hepatic SULT1A1 protein levels and SULT1A1 gene copy numbers were determined: the ME DNA adduct level positively correlated with the SULT1A1 protein expression and SULT1A1 gene copy numbers (*p* = 6.6 × 10^–7^ and 3 × 10^–3^, respectively).

In that study, several other absorption, distribution, metabolism, and elimination (ADME) mRNAs were correlated with the DNA adduct levels positively (including SULT1E1, SULT1A2, CYP1A1, and CYP1A2) or negatively (including GSTP1) in the primary analysis, but all these correlations were absent or lost their statistical significance after adjustment for the impact of SULT1A1 mRNA (Supporting Information of Tremmel et al.^17^). Indeed, after this adjustment, no correlation was observed between the adduct levels and the mRNA levels of any xenobiotic metabolizing enzymes (trivially except SULT1A1). The list of the enzymes studied contained 47 CYPs (including CYP1A2, 2B6, and 2C9), 9 UGTs (including UGT2B7), and 18 cytosolic and microsomal GSTs (including GSTT1). However, it must be taken into account that these correlation analyses were conducted using the expression levels at the time of surgery, whereas the DNA adducts may have been formed at much earlier times. For example, a substantial level of the DNA adducts formed by safrole in the mouse liver was still present 140 days after the treatment.^15^ These temporary differences may be particularly important for enzymes whose expression is primarily determined by enzyme induction (e.g., CYP1A2 and 2B6) rather than genetic factors (which are important, e.g., with SULT1A1, GSTT1, GSTM1). Thus, patients may have changed their lifestyle and received new drug treatments in the period before surgery. Therefore, the critical role of the levels of CYP1A2 and 2B6 in the activation of ME cannot be strictly excluded on the basis of this study.

### Significance of Urinary *E*-3′-MEMA as a Biomarker for ME Exposure, Activation, and Detoxification

In the current study, formation and excretion of MEMA in human urine after consumption of ME-containing food was demonstrated. In total, three isomers of MEMA could be detected in the pilot investigation. However, only the most abundant MEMA isomer, *E*-3′-MEMA, was excreted in traceable amounts in the main study, involving a lower exposure than in the pilot investigation. The chemical structure of *E*-3′-MEMA was proven by comparison with a selectively synthesized standard. To the best of our knowledge, besides DNA adducts of ME, no further reaction products of metabolically activated ME have been reported in humans so far. In this context, *E*-3′-MEMA can be considered the first noninvasive biomarker for ME exposure and activation in humans.

However, the application as a potential biomarker for dietary exposure to ME (*reverse dosimetry*) is disputable. On the one hand, *E*-3′-MEMA is very specific for the exposure to ME, i.e., there is no other known source of this metabolite. On the other hand, it is unfavorable that only a very small portion of ME is excreted as *E*-3′-MEMA. This would have to be compensated for by a very sensitive mass spectrometric method. However, the conversion ratios between 1 and 85 ppm indicate that previously estimated daily ME exposures, e.g., 1–10 μg/kg body weight^61^ hardly lead to detectable *E*-3′-MEMA concentrations in the urine. Also, in the current study, *E*-3′-MEMA was hardly detectable after 12 h after ME exposure. The second complication is the high interindividual variability of *E*-3′-MEMA excretion observed here (with a factor of ∼80 between the highest and lowest values), reflecting the interindividual differences in the metabolism of ME between the study participants. High variation in the urinary excretion of a congeneric mercapturic acid was also observed after the controlled exposure of volunteers to estragole and *trans*-anethole taken up in 500 mL fennel tea.^37^ Because of these differences, it is highly imprecise to draw conclusions from daily *E*-3′-MEMA excretion about daily ME intake.

However, the detection of a mercapturic acid in urine implies that the subject was exposed to the corresponding compound (ME in our study) and that the compound was bioactivated and detoxified. In general, a high level of a mercapturic acid in a subject may be due to high exposure, extensive bioactivation, and/or efficient detoxification of the reactive intermediate. The two latter factors that can lead to high levels of urinary mercapturic acids have opposing effects on individual risk. Therefore, the individual risk cannot be estimated from the mercapturic acid level without additional information. In the case of ME, only a minute fraction of the dose was excreted as MEMA, arguing against a relevant role of the mercapturic acid pathway in the detoxification of ME. Moreover, the comparable pattern of MEMA isomers in the pilot investigation with that of the chemically prepared MEMA mixture, and lack of associations between GST expression and ME DNA adduct levels in human liver samples^17^ also suggest that the conjugation of reactive ME intermediates with GSH (and/or other forms of cysteine) occurs nonenzymatically and therefore with only little interindividual variability. Thus, we postulate that individuals (or circumstances) with high urinary MEMA levels reflect high exposure to the reactive ME intermediate (resulting from high ME exposure, particularly effective bioactivation, or both) rather than efficient detoxification, a hypothesis to be corroborated in further investigations.

## Supporting Information Available

Methyleugenol and eugenol contents in basil cultivars; recipe of the basil pesto used for the controlled exposure study; product ion mass spectra of peaks 1-3 in the MEMA isomer mixture;^1^H NMR, ^13^C NMR and MS spectrum of synthesized *E*-3'-MEMA; LC–MS characterization of synthesized *d*_6_-MEMA; GC–MS/MS characterization of ME and *d*_3_-ME; GC–MS/MS analysis of ME in basil leaves and pesto; serial dilution of *d*_6_-*E*-3'-MEMA in water or urine matrix; and details of initial GC–MS analysis for ME determination (PDF)
