# Supplementary material for: Urinary Excretion of Mercapturic Acids of the Rodent Carcinogen Methyleugenol after a Single Meal of Basil Pesto: A Controlled Exposure Study in Humans
Source: Chem Res Toxicol. 2023 Oct 24;36(11):1753–67. doi: 10.1021/acs.chemrestox.3c00212 (PMC10664145; doi:10.1021/acs.chemrestox.3c00212)
Supplement: Supplementary file 1 — tx3c00212_si_001.pdf [file tx3c00212_si_001.pdf]

Supplemental Material

Urinary excretion of mercapturic acids of the rodent carcinogen methyleugenol after a single meal of basil pesto – a controlled exposure study in humans

Kai Nieschalke[a,b,#], Nick Bergau[b,#], Sönke Jessel[d], Albrecht Seidel[d], Susanne Baldermann[e,f], Monika Schreiner[e], Klaus Abraham[b], Alfonso Lampen[b], Bernhard H. Monien[b], Burkhard Kleuser[a,c], Hansruedi Glatt[b,*], Fabian Schumacher[a,c,*]

[a] Department of Nutritional Toxicology, Institute of Nutritional Science, University of Potsdam,14558 Nuthetal, Germany

[b] German Federal Institute for Risk Assessment (BfR), Department of Food Safety, 10589 Berlin, Germany

[c] Department of Pharmacology and Toxicology, Institute of Pharmacy, Freie Universität Berlin, 14195 Berlin, Germany

[d] Biochemical Institute for Environmental Carcinogens, Prof. Dr. Gernot Grimmer-Foundation, 22927 Grosshansdorf, Germany

[e] Department Plant Quality and Food Security, Leibniz Institute of Vegetable and Ornamental Crops (IGZ), 14979 Grossbeeren, Germany

[f] Faculty of Life Sciences: Food, Nutrition & Health, University of Bayreuth, 95326 Kulmbach, Germany

[#] authors contributed equally

[*] to whom the correspondence should be addressed

Corresponding author email addresses:

glatt@dife.de (HG) and fabian.schumacher@fu-berlin.de (FS)

Table of Contents

Table S1. Methyleugenol and eugenol contents in basil cultivars S3

Table S2. Recipe of the basil pesto used for the controlled exposure study S4

Figure S1: Product ion mass spectra of peaks 1-3 in the MEMA isomer mixture S5

Figure S2: ^{1}H NMR, ^{13}C NMR and MS spectrum of synthesized *E*-3'-MEMA S6

Figure S3: LC-MS characterization of synthesized d_6-MEMA S7

Figure S4: GC-MS/MS characterization of ME and d_3-ME S8

Figure S5: GC-MS/MS analysis of ME in basil leaves and pesto S9

Figure S6: Serial dilution of d_6-*E*-3'-MEMA in water or urine matrix S10

Methods: Details of initial GC-MS analysis for ME determination S11

Tables

Table S1. Methyleugenol and eugenol content [µg/g fresh weight, mean ± SEM] in cultivars used for the screening experiment.

Name of cultivar	Methyleugenol	Eugenol
Genoveser	138.2 ± 26.3	309.8 ± 20.3
Greek Basil	75.7 ± 10.0	290.7 ± 48.2
Provence	40.6 ± 27.0	41.1 ± 11.2
Rosie	51.8 ± 10.9	251.8 ± 28.4
Red Genoveser	24.3 ± 2.2	516.9 ± 19.5
Lemon basil (small-leaved)	0.5 ± 0.0	17.1 ± 1.2
African lemon	0.6 ± 0.1	17.0 ± 1.5
Cinnamon basil	7.1 ± 4.9	69.3 ± 53.2
Thai basil - Siam Queen	11.1 ± 1.8	14.6 ± 0.2
Thai-Basil - Purple Crown	6.1 ± 0.8	14.4 ± 0.6
Thai-Basil - Green Joy	117.2 ± 41.3	14.1 ± 0.2
African spice	0.5 ± 0.1	13.7 ± 0.2
Neu Guinea	4.6 ± 0.6	13.7 ± 0.3
Peru basil	128.3 ± 41.7	1063.0 ± 58.8
Madeira basil	3.1 ± 0.3	13.4 ± 0.3
Bavita	1.0 ± 0.3	407.9 ± 36.6
Red Basil Rosie	93.8 ± 17.2	249.3 ± 32.7
Great green	1.7 ± 0.9	358.4 ± 58.4

Table S2. Recipe of the basil pesto used for the controlled exposure study in humans. The listed ingredients resulted in a total quantity of ~332 g pesto. For the human intervention study, a total quantity of 3 kg basil pesto was produced by scaling up.

Ingredient	Amount
Basil leaves	100 g
Pine nuts	50 g
Grana Padano	50 g
Olive oil	150 mL (≙ 132 g)

Figures

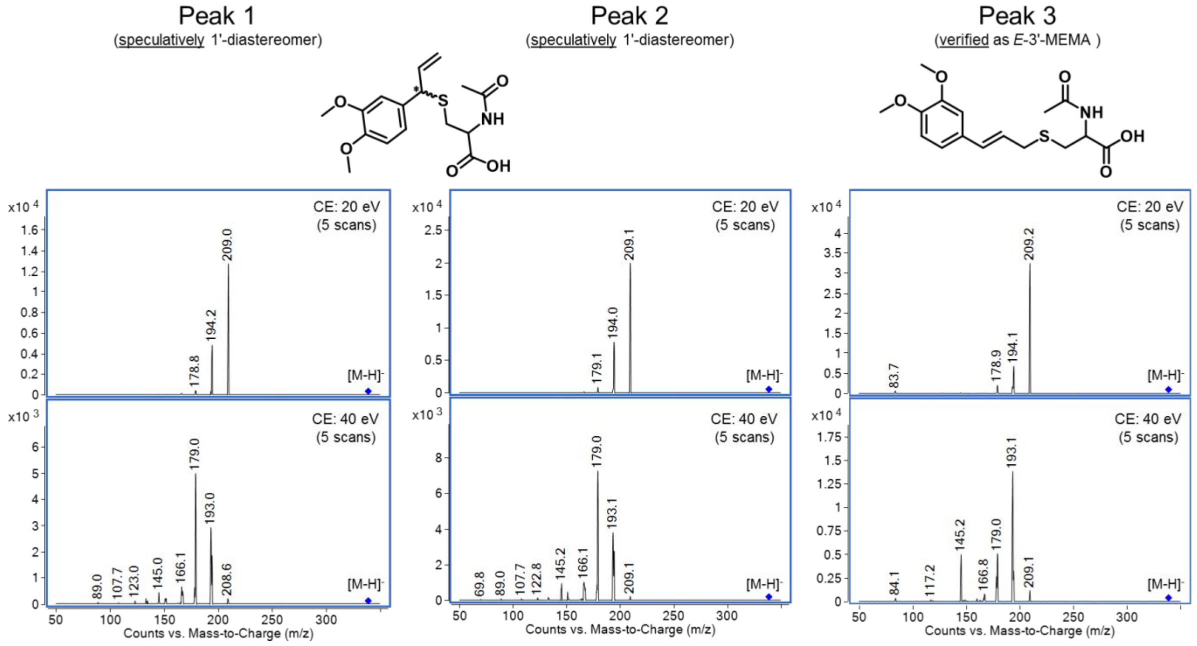

Figure S1: Product ion mass spectra of Peaks 1-3 in the MEMA isomer mixture (see Figure 1, main document) recorded at 20 eV (upper spectra) or 40 eV (lower spectra) collision energies (CE). Peak 3 was verified as *E*-3'-MEMA (see Figure S2). Relative intensities of fragment ions in the 40 eV-MS/MS spectra differ substantially for Peak 3 (base peak *m/z* 193.1) compared to Peaks 1 & 2 (base peak *m/z* 179.0). Peaks 1 & 2 are presumably a pair of diastereomers after 1'-conjugation (assumed chemical structure is given as inset).

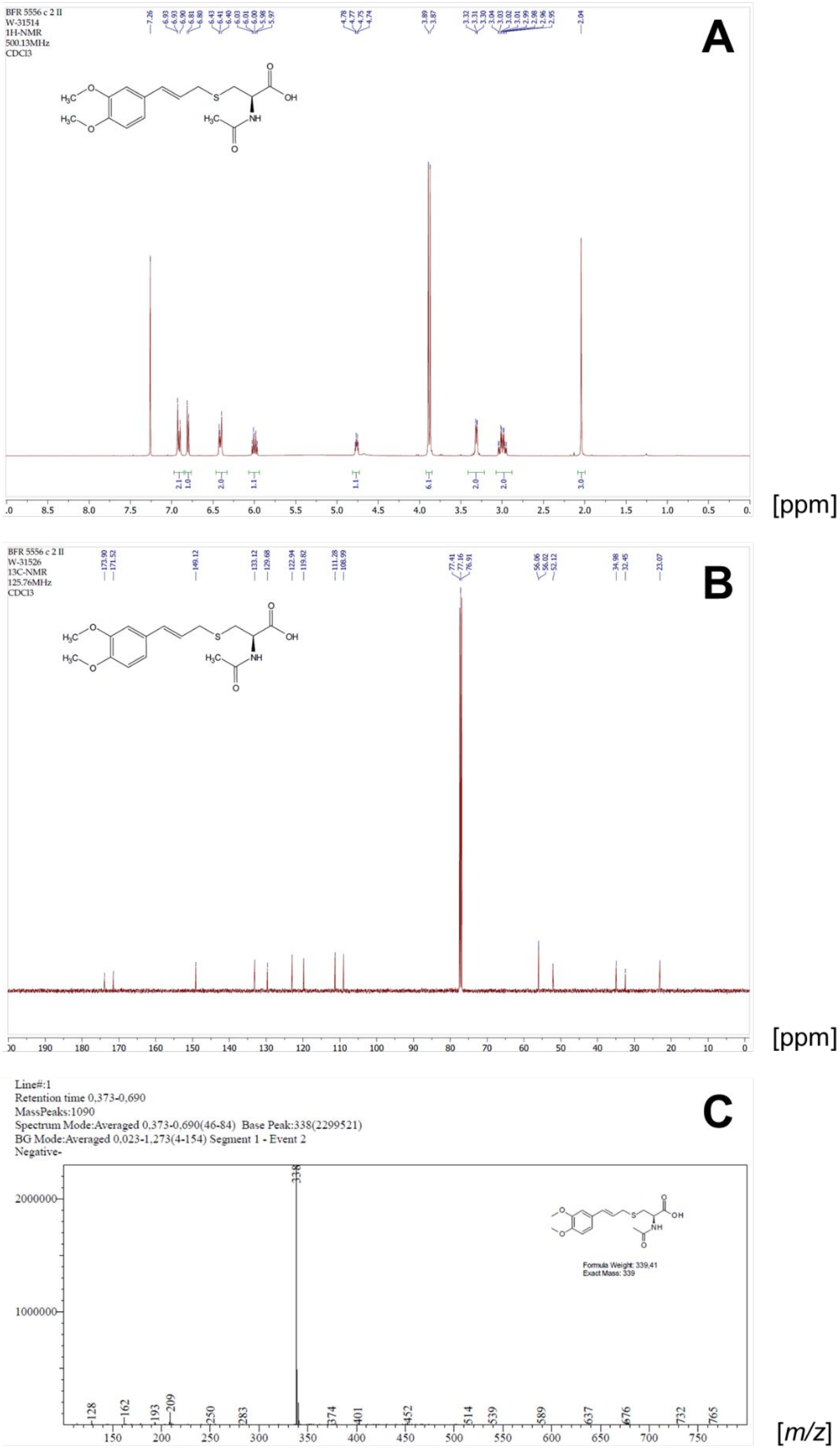

Figure S2: (A) ^{1}H NMR, (B) ^{13}C NMR and (C) MS spectrum of synthesized *N*-acetyl-*S*-[3'-(3,4-dimethoxyphenyl)allyl]-L-cysteine (*E*-3'-MEMA).

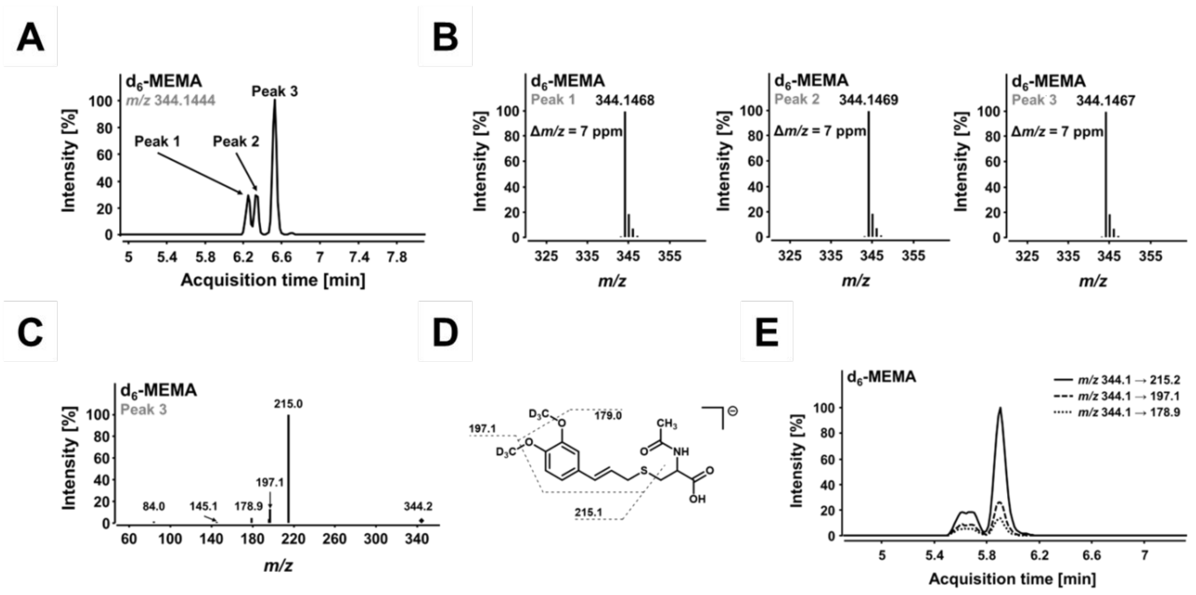

Figure S3: LC-MS characterization of synthesized d_6-MEMA using instrumental set-up "system 1". (A) High-resolution mass spectrometry (HRMS) chromatogram of d_6-MEMA in ESI- single ion monitoring (SIM) mode. Three prominent signals (Peaks 1, 2, and 3) were detected at *m/z* 344.1444. (B) Isotopic pattern of isomeric d_6-MEMA Peaks 1-3 and corresponding mass error (Δ*m/z*). (C) Product ion mass spectrum of Peak 3 obtained at a collision energy of 20 eV. The associated precursor ion was set at *m/z* 344.2. (D) Chemical structure of d_6-*E*-3'-MEMA and suspected fragmentation pattern. (E) Multiple reaction monitoring (MRM) chromatogram of d_6-MEMA. An overlay of multiple transitions is given.

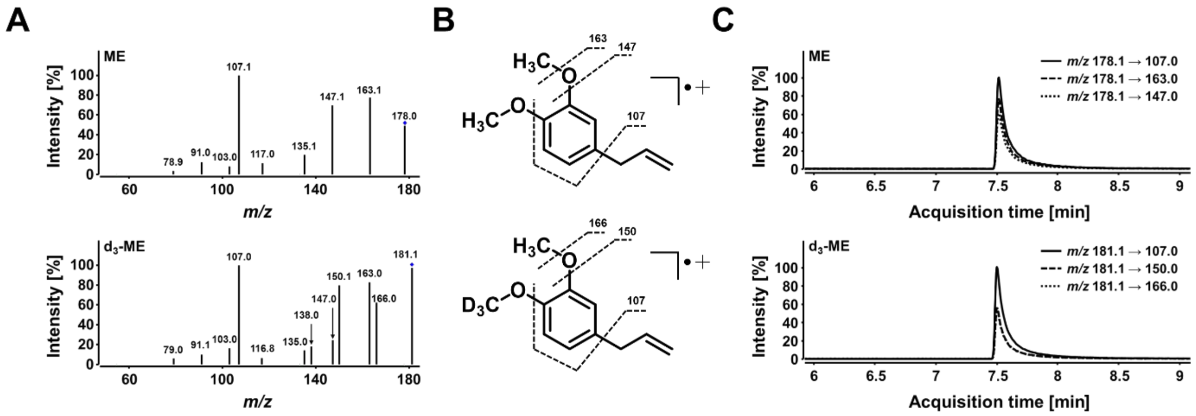

Figure S4: GC-MS/MS characterization of ME (upper panel) and d_3-ME (lower panel). (A) Product ion mass spectrum of ME and d_3-ME obtained at a collision energy of 10 eV. The associated precursor ion was set at *m/z* 178 and 181, respectively. (B) Chemical structure of ME and d_3-ME including the presumed fragmentation pattern for the three most prominent product ions. (C) Multiple reaction monitoring (MRM) chromatogram for ME and d_3-ME. An overlay of the different transitions used for analysis is shown.

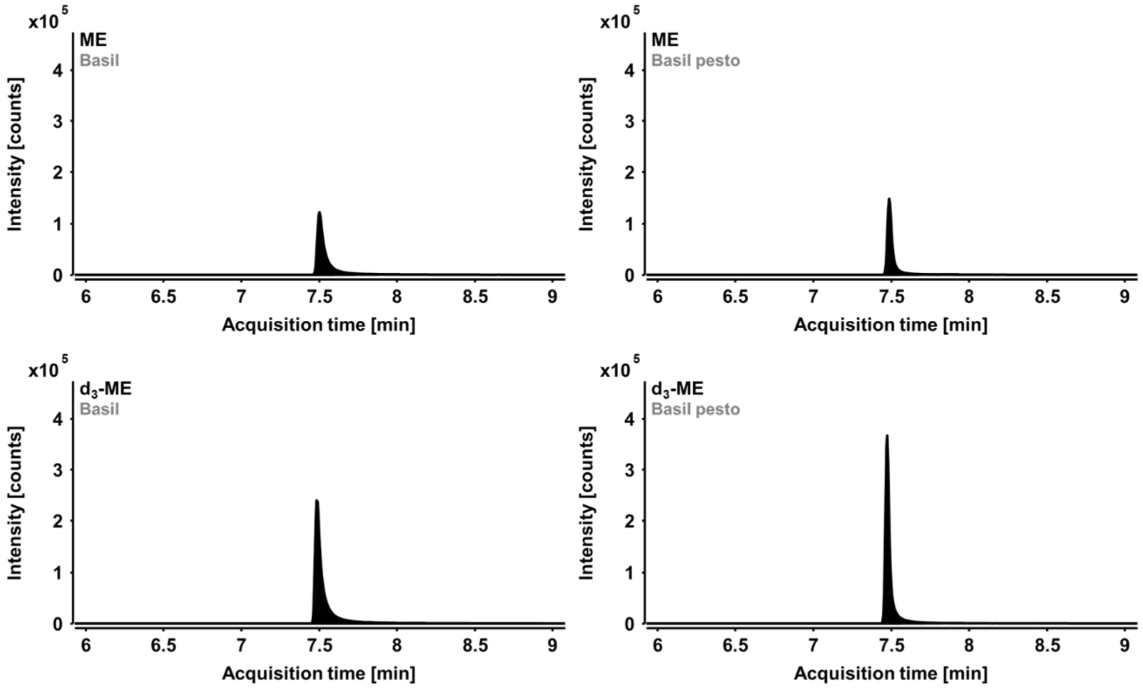

Figure S5: GC-MS/MS analysis of ME in basil leaves and in pesto made from them. Shown are the quantifier mass transitions of ME (upper panel; *m/z* 178.1 → 107.0) and d_3-ME (lower panel; *m/z* 181.1 → 107.0). Corresponding signals of ME and its internal standard are shaded black.

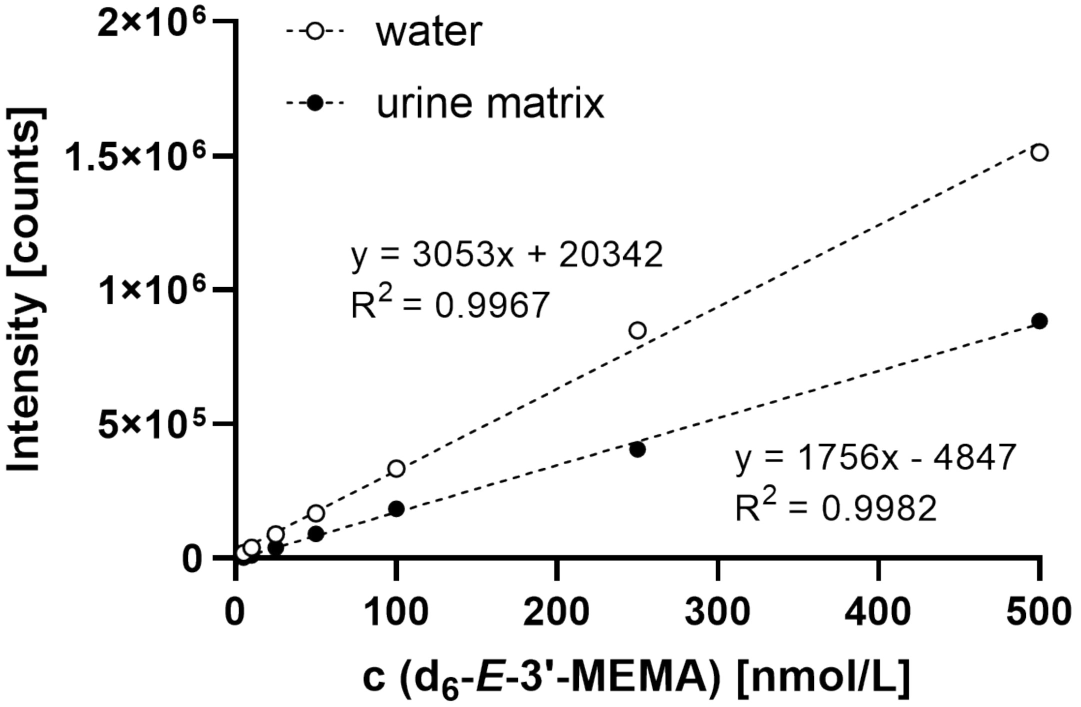

Figure S6: Serial dilution of d_6-*E*-3'-MEMA in water (open circles) or urine matrix (closed circles) measured with the optimized LC-MS/MS method ("system 2"), which was applied to urine samples of the intervention study including twelve participants. Equations of linear regression together with coefficients of determination are given as insets.

Methods

Details of GC-MS analysis to identify the basil cultivar with the highest methyleugenol content

The GC-MS system consisted of an Agilent 7890B GC-System (Agilent Technologies, Waldbronn, Germany) coupled to an Agilent 7010 triple-quadrupole mass spectrometer (Agilent Technologies) and equipped with a Multi-Purpose-Sampler (MPS, Gerstel, Mülheim an der Ruhr, Germany). The MPS injection system was operated with the following temperature program: 40 °C for 3 min, increase of 2 °C/min until 60 °C, and hold for 2 min, then increase of 3 °C/min until 180 °C, and hold for 10 min isothermally. The cryofocusing program started at –100 °C. Then the temperature was increased at 12 °C/min to 270 °C and then maintained at 270 °C for 3 min. Twisters desorption was performed with a Gerstel Thermal Desorption Unit (TDU) with the following temperature program (starting temperature, 25 °C; increase of 720 °C/min until 270 °C, hold for 8 min at 270 °C). The MS analysis was carried out in a full-scan mode with a scan range of *m/z* 50-300. For chromatography, helium (5.0) with a constant flow rate of 1.2 mL/min was used as carrier gas. The temperature for the inlet was 280 °C and separation achieved on a HP-5MS column (0.25 mm x 30 m, 0.25 µm; Agilent Technologies) using the following temperature program: starting at 40 °C (held for 2 min), increased to 300 °C at a rate of 5 °C/min (held for 5 min). Quantification was performed *via* external calibration curves using eugenol and methyleugenol as reference compounds.
